# The price and affordability of essential medicines, progress and regional distribution in China: a systematic review

**DOI:** 10.3389/fphar.2023.1153972

**Published:** 2023-05-05

**Authors:** Zheng Liu, Kun Zou, Dan Liu, Miao Zhang, Yuqing Shi, Zhe Chen, Bingchen Lang, Xiao Cheng, Hailong Li, Linan Zeng, Yong Tang, Shaoyang Zhao, Imti Choonara, Yongmu Jiang, Lingli Zhang

**Affiliations:** ^1^ Department of Pharmacy, West China Second University Hospital, Sichuan University, Chengdu, China; ^2^ Evidence-Based Pharmacy Center, West China Second University Hospital, Sichuan University, Chengdu, China; ^3^ NMPA Key Laboratory for Technical Research on Drug Products *In Vitro* and *In Vivo* Correlation, Chengdu, China; ^4^ Key Laboratory of Birth Defects and Related Diseases of Women and Children, Sichuan University, Ministry of Education, Chengdu, China; ^5^ West China School of Medicine, Sichuan University, Chengdu, China; ^6^ West China School of Pharmacy, Sichuan University, Chengdu, China; ^7^ School of Economics, Sichuan University, Chengdu, China; ^8^ School of Medicine, University of Nottingham, Nottingham, United Kingdom; ^9^ Chinese Evidence-Based Medicine Center, West China Hospital, Sichuan University, Chengdu, China

**Keywords:** affordability, China, essential medicines, median price ratio, systematic review

## Abstract

**Background:** Essential medicine is a vital component to assure universal access to quality healthcare. However, the trend of affordability to essential medicines in China and its regional differences were not yet fully understood. This study aimed to systematically evaluate the price and affordability of essential medicines, their progress, and regional distribution in China in the last decades.

**Methods:** We searched seven databases and three websites for potentially eligible studies from inception until March 2022. Studies on the price and affordability of essential medicines investigated in China were included. Median and interquartile range (IQR) was used to describe the price and affordability of essential medicines, and compared in three periods, before 2009, from 2009 to 2014, and from 2015 to 2019. Subgroup analysis was performed to examine the price and affordability by regions, health facilities, and ATC categories of medicines. The study was registered with PROSPERO (CRD42022310173).

**Results:** A total of 65 studies including 11,639 health facilities investigated between 2006 and 2019 were included in this review. Median price ratios (MPR) and affordability of essential medicines were reported in 44 studies and 50 studies, respectively. The median MPRs of essential medicines in China was 1.59 (IQR: 5.39), with a tendency to rise first and then fall from 2006 to 2019. And the median affordability was equal to 0.88 (IQR: 2.58) days’ wage of the lowest paid unskilled government worker, but steadily rose from 2006 to 2019. Subgroup analysis showed that the affordability in the western region (1.40, IQR: 2.88), urban area (0.95, IQR: 2.80), private sector (0.90, IQR: 2.30), of originator brands (OB) (2.90, IQR: 6.68), and antineoplastic and immunomodulating agents (5.68, IQR: 56.47) were worse than their counterparts.

**Conclusion:** The prices of essential medicine were higher than international level, the overall affordability of essential medicines in China is acceptable but poor in the western region, for OB drugs and anti-cancer medicines. Further national essential medicine policies are needed to reduce regional disparities and improve the affordability of expensive drugs.

**Systematic Review Registration:** https://www.crd.york.ac.uk/PROSPERO/#recordDetails

## 1 Introduction

Essential medicines are those that satisfy the priority healthcare needs of the population and should be available in the context of functioning healthcare systems. It should be in sufficient supply, appropriate dosage forms, consistent quality, adequate information, and at a price that the individual and the community can afford (World Health Organization, 2002). Access to safe, effective, quality and affordable essential medicines and vaccines for all to achieve universal health coverage is one of the Sustainable Development Goals (SDGs 3.8) of the United Nations (Sustainable Development, 2020). However, according to the report by World Health Organization (WHO) (World Health Organization, 2017), it is estimated that nearly two billion people have no access to essential medicines, causing a cascade of misery and suffering that can be easily and inexpensively prevented or treated through simple and affordable interventions. It was reported that about 10 million lives a year could be saved by the implementation of the National Essential Medicines Policy (World Health Organization, 2008).

WHO had published the first model list of essential medicines in 1977 and revised it every 2 years for adapting to the circumstances in more than one hundred countries. Meanwhile, A standard methodology for measuring medicine prices, availability, and affordability was developed by the WHO and Health Action International, 2021 (World Health Organization, 2008) and has been applied in more than 60 countries. (HAI) However, global access to essential medicines is facing formidable challenges. A survey investigated in 25 countries between 2008 and 2019 showed a substantial variation in access, with a range of 0%–69% (median of 15%) of health facilities providing an available and affordable set of essential medicines for the treatment, prevention, and management of acute and chronic diseases, and no facility offered all these essential medications in over one-quarter (28%) of the nations (World Health Organization, 2022). The price barrier was the main reason hindering access to essential medicines. The high price and cost of medicines have impaired the ability of many healthcare systems to provide population-wide access (World Health Organization, 2020). Especially, in the absence of insurance coverage, patients and their families often experience significant financial burdens when in need of medications (Cohen and Kirzinger, 2014).

As in many developing countries, the lack of access to essential medicines has caused growing concern in China and a series of measures have been taken to meet the public’s basic healthcare needs. Since 1982, China had officially issued the first edition of the national essential medicine list (NEML) and revised it eight times. The latest edition was updated in 2018 and involved 685 medicines, among which the top three medicines in the highest proportion are used for cardiovascular diseases, endocrine diseases, and anti-microbial (Zuo et al., 2020). Up to now, several studies were carried out to evaluate the price and affordability of essential medicines across China, which found that the affordability of some medicines was still challenging (Zhu et al., 2019; Dong et al., 2020; Wang et al., 2021). However, most studies focused on specific regions or provinces at a single time point. Long-term national monitoring and evaluation of the progress of affordability of essential medicines in China are lacking, and its geographic variations among regions and provinces are unclear. Thus, this study aimed to systematically evaluate the price and affordability of essential medicine, their progress and regional distribution in China, to inform evidence-based policy-making to improve the affordability and equitable access to essential medicines in China.

## 2 Materials and methods

This systematic review was reported in accordance with the Preferred Reporting Items for Systematic Review and Meta-Analysis (PRISMA), and was registered at the International Prospective Register of Systematic Reviews (CRD42022310173).

### 2.1 Search strategy and selection criteria

We searched PubMed, EMBASE (Ovid), CENTRAL (Ovid), Web of Science (WOS), The Cochrane Library, China National Knowledge Infrastructure (CNKI), Chinese BioMedical Literature Database (CBM), and the official websites of World Health Organization (WHO), International Pharmaceutical Federation (FIP), and Health Action International, 2021 from inception until 1 March 2022. We used the following combined text and MeSH terms: “essential medicine*” and “China.” The detailed search strategies were provided in Supplementary Appendix SA1. Additionally, the reference lists of included studies were reviewed for more eligible studies. The language was restricted to English and Chinese.

We included studies if they investigated the affordability of essential medicines in hospitals, pharmacies, or drug supply enterprises in China; were cross-sectional studies, interrupted time series studies, controlled or uncontrolled before-after studies; reported outcomes of median price, median price ratios (MPR), or affordability. A study was excluded if it was a duplicate publication or the full text was not available. Based on WHO/HAI standard methodology, the MPR is defined as the ratio of the median local unit price of a medication to the median international reference unit price, in which the price of a medicine is lower than the international level if the value was less than 1; and the affordability is defined as the ratio of the monthly treatment cost of a medication to the daily wage of the lowest paid unskilled government worker, in which a medicine is considered affordable if the value was less than 1 (World Health Organization, 2008).

### 2.2 Study selection and data extraction

Two reviewers (ZL and MZ) independently screened the title and abstract for potential studies in available records using Endnote X9 (Clarivate Analytics, United Kingdom), and the final included studies were determined after reading the full text according to the inclusion criteria.

A standardized table was designed for extraction and a pre-test with 10% of the included studies was conducted to revise the table and formulate instructions for filling it. The following information of included studies was independently extracted using Microsoft Excel (Microsoft Corporation, United States) by two reviewers (ZL and MZ): i) basic information of literature including title, first author, publication year, and study design; ii) research details including data collection period, investigation region (eastern/central/western/northeastern region, and province Supplementary Appendix SA2), area (urban/rural), health facility (public/private, tertiary/secondary/primary hospital, pharmacy or enterprise), sample size, methodology, and medicine list (generic name, characteristic and category of medicine); iii) outcome measures including median prices, MPR, and affordability. Disagreements were resolved through discussion with the third reviewer (KZ).

### 2.3 Risk of bias assessment

According to the Joanna Briggs Institute (JBI) appraisal tools, two reviewers (ZL and MZ) evaluated the methodological quality and risk of bias of studies included in our systematic review independently. The checklist consists of 9 items across four domains: i) sampling methods; ii) research objects; iii) data collection, and iv) analysis methods (Munn et al., 2015). Each item was rated “yes” (one point), “no” (zero points), “unclear” (zero points), or “not applicable” (one point), and the methodological quality of each study was graded into low (0–3), moderate (4–6), and high (7–9) (Wilairatana et al., 2021). Disagreements were resolved by consulting the third reviewer (KZ).

### 2.4 Statistical analysis

Descriptive statistics were conducted, in which the summary measures of the price and affordability reported in included studies were expressed as median and interquartile range (IQR). We examined the price and affordability of essential medicines in three periods based on the time of the survey, namely, before 2009, from 2009 to 2014, and from 2015 to 2019, to explore the changes over time before and after the implementation of national essential medicines policy. Then, we performed subgroup analysis, if the data available, to further examine the price and affordability of essential medicines during three periods by: i) regions (eastern, central, western, or northeastern region); ii) provinces; iii) areas (urban or rural); iv) types of health facilities (public or private sector); v) levels of health facilities (tertiary, secondary or primary); vi) characteristic of medicines [originator brand (OB) or lowest priced generic equivalent (LPG)]; and vii) category of medicines of the anatomic therapeutic chemistry (ATC) classification. Statistical analyses were performed using Stata, version 17.0 (Stata Corp., United States).

## 3 Results

### 3.1 Search results

**FIGURE 1:**
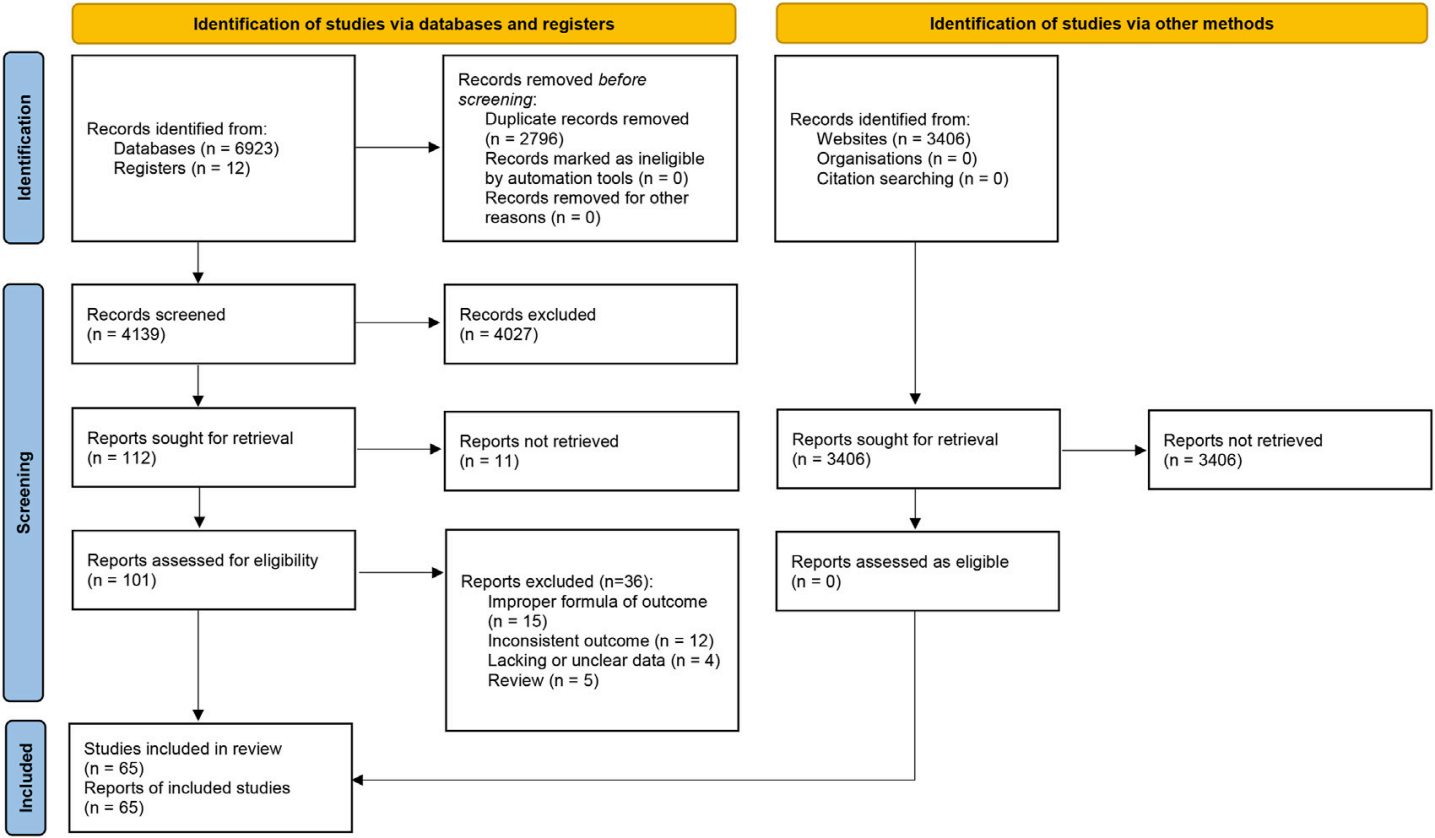
PRISMA flow diagram of study selection.

We identified 6,935 citations from seven databases and registers and 3,406 records from three websites. After the removal of duplicates (*n* = 2796), 4,139 titles and abstracts were screened, 112 of which were selected for full-text review. The records from the websites were also screened but no eligible study was found. Following the assessment of 101 potentially eligible articles, 65 studies were eventually included in this review. The flow diagram of study selection is shown in Figure 1.

### 3.2 Characteristics of included studies and risk of bias

The included 65 studies were all cross-sectional studies, involving a total of 11,639 health facilities (except nine studies that not reported the number of investigated health facilities), and were conducted from 2006 to 2019. Of these 65 studies, 25 studies (38.46%) were conducted in eastern China, 13 (20.00%) were in central China, 14 (21.54%) were in western China, 2 (3.08%) were in the northeastern region, and 12 studies (18.46%) were cross-regional survey. Among them, 23 studies (35.38%) investigated in urban areas, 9 studies (13.85%) investigated in rural areas, and 33 studies (50.77%) investigated both urban and rural areas simultaneously. As regard survey methods, 61 studies (93.85%) adopted the WHO/HAI standardized methodology to investigate the affordability of essential medicines, while 4 studies (6.15%) used non-WHO/HAI methods but adopted the same definition of measured outcomes. The detailed characteristics of the included studies are shown in Supplementary Appendix SA4 and the number of reported studies of each subgroup is shown in Supplementary Appendix SA5 in detail.

Among the 65 studies, 12 studies attained a score of 8, 12 studies a score of 7, 14 studies a score of 6, 9 studies a score of 5, 13 studies a score of 4, 4 studies a score of 3, and 1 study a score of 2. Overall, 24 studies (36.92%) were assessed as high quality, 36 studies (55.38%) were of moderate quality, and 5 studies (7.69%) were of low quality. The results of the quality assessment in this review are presented in Supplementary Appendix SA6.

### 3.3 Price of essential medicines in China

**FIGURE 2:**
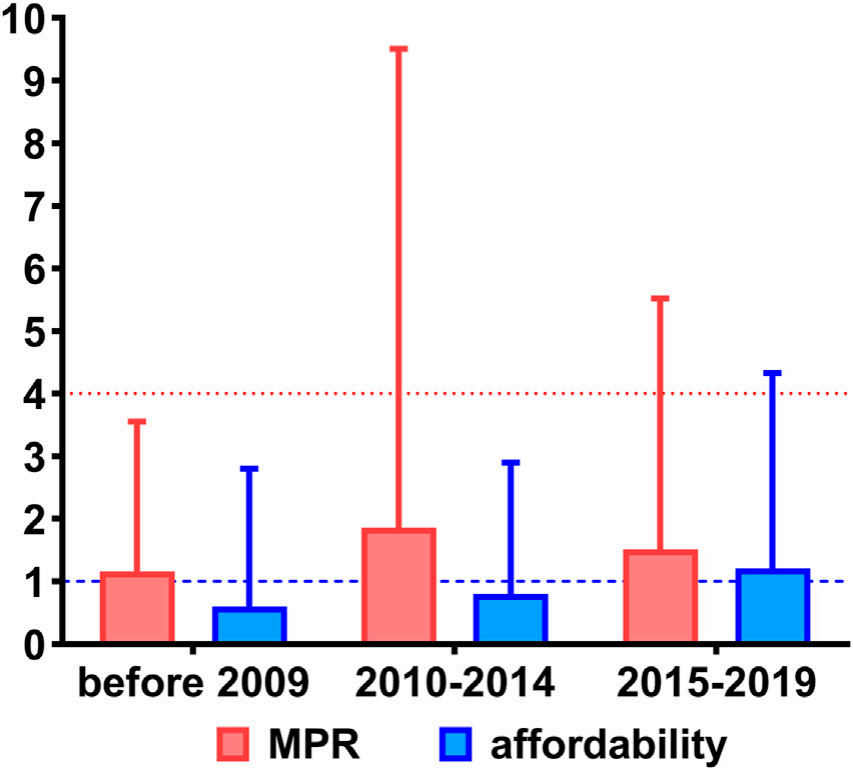
The overall price and affordability of essential medicines in China. Notes: Red indicates the MPRs of essential medicines with a red dotted line as the threshold, and blue indicates the affordability of essential medicines with a blue dotted line as the threshold. MPR, median price ratio.

A total of 44 studies reported the MPR of essential medicines. The median MPRs of essential medicines nationwide of all included studies was 1.59 (IQR: 5.39), with the trend of rising first and then falling before 2009 (1.15, IQR: 2.40), from 2010 to 2014 (1.86, IQR: 7.65), and from 2015 to 2019 (1.51, IQR: 4.01). The overall trend of the prices is presented in Figure 2.

**FIGURE 3:**
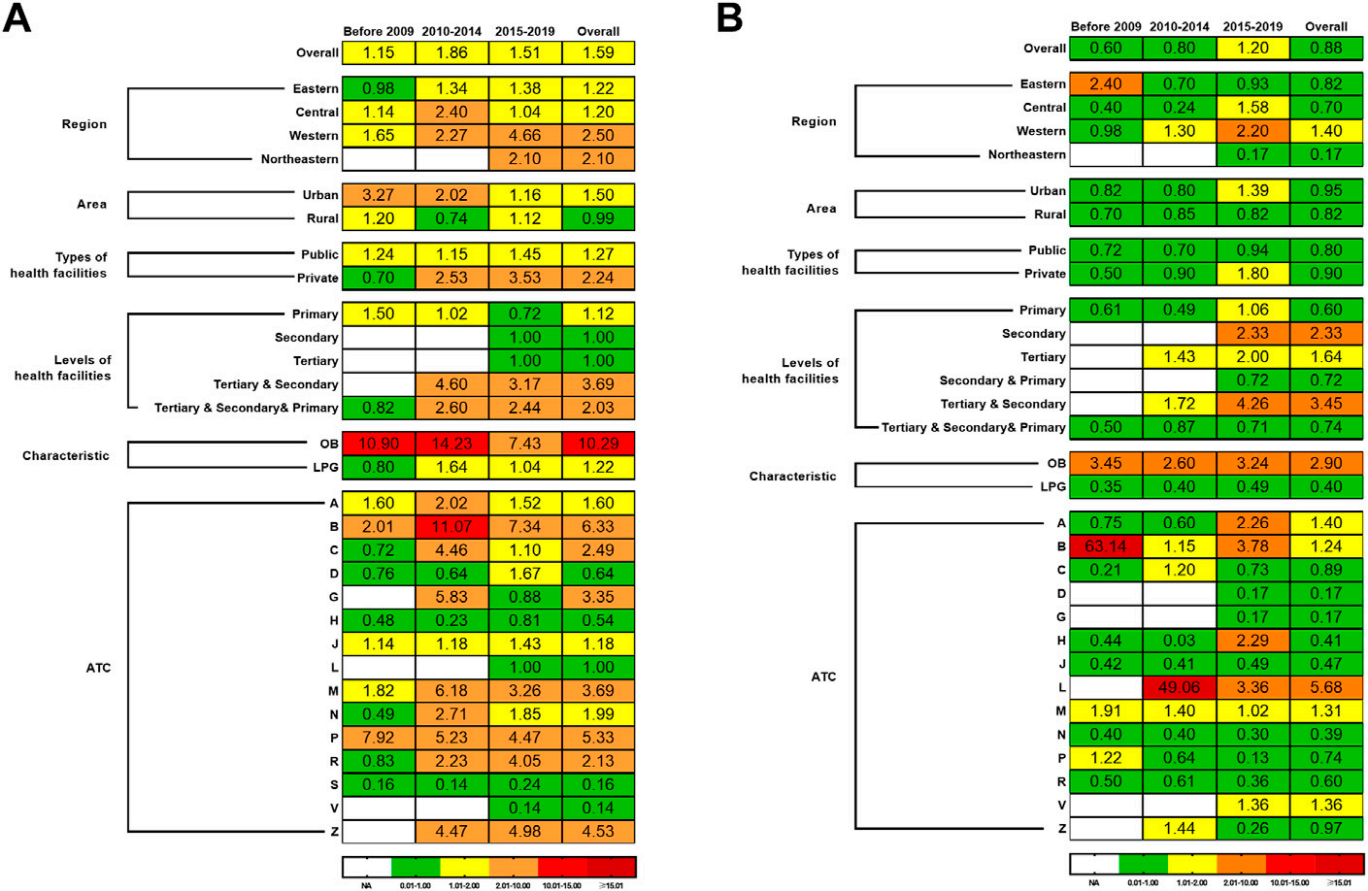
The price and affordability of essential medicines in China. Notes: **(A)** the median MPRs of essential medicines in China; **(B)** the median affordability of essential medicines in China. MPRs, median price ratios; OB, originator brand; LPG, lowest priced generic equivalent; ATC, anatomic therapeutic chemistry; A, alimentary tract and metabolism; B, blood and blood-forming organs; C, cardiovascular system; D, dermatologicals; G, genito urinary system and sex hormones; H, systemic hormonal preparations, excl. sex hormones and insulins; J, antiinfectives for systemic use; L, antineoplastic and immunomodulating agents; M, musculoskeletal system; N, nervous system; P, antiparasitic products, insecticides and repellents; R, respiratory system; S, sensory organs; V, various; Z, unknow.

**FIGURE 4:**
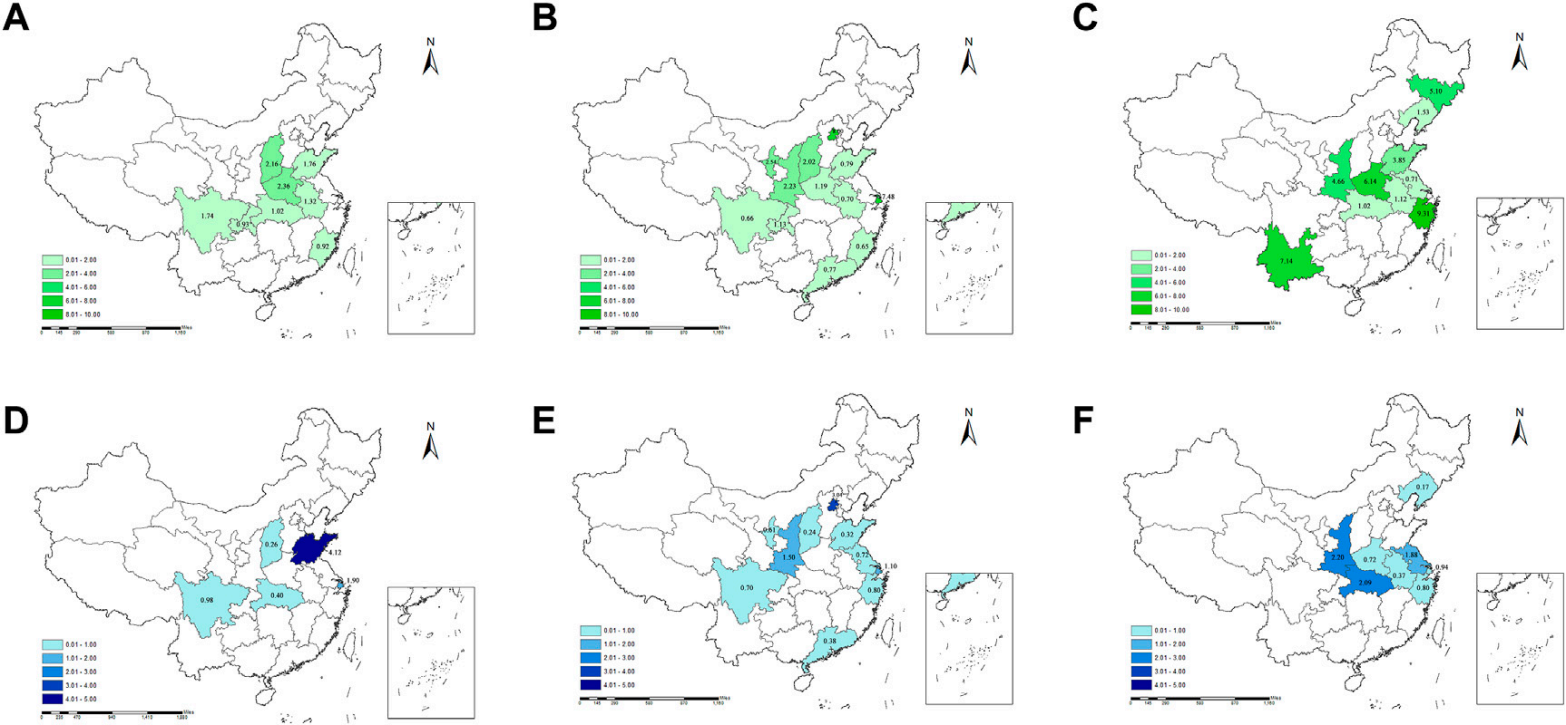
The provincial characteristics of the price and affordability of essential medicines in China. Notes: i) the upper pictures show the MPRs of essential medicines of each province in China before 2009 **(A)**, from 2010 to 2014 **(B)**, and from 2015 to 2019 **(C)**; ii) the bottom pictures show the affordability of essential medicines of each province in China before 2009 **(D)**, from 2010 to 2014 **(E)**, and from 2015 to 2019 **(F)**. MPRs, median price ratios.

Subgroup analysis showed that medicine prices in the western region (2.50, IQR: 8.29) were higher than in other regions. It varied largely from the highest in Zhejiang province (9.31, IQR: 9.06) to the lowest in Jiangsu province (0.71, IQR: 2.52). The MPRs of urban area (1.50, IQR: 4.03), private sector (2.24, IQR: 6.97), tertiary and secondary health facilities (3.69, IQR: 5.17), and OB drugs (10.29, IQR: 18.68) were higher than their counterparts. The top three ATC types of essential medicines with the highest prices were blood and blood-forming organs drugs (B) (6.33, IQR: 17.15), antiparasitic products, insecticides and repellents (P) (5.33, IQR: 5.93), and musculoskeletal system drugs (M) (3.69, IQR: 17.84). The results of subgroup analysis of the prices are presented in Figure 3A, among which the provincial characteristics are shown in Figures 4A–C. The detailed results are listed in Supplementary Appendix SA7.

### 3.4 Affordability of essential medicines in China

A total of 50 studies reported the affordability of essential medicines. The median affordability of essential medicines nationwide was equal to 0.88 (IQR: 2.58) days’ wage, with the trend of gradual growth before 2009 (0.60, IQR: 2.20), from 2010 to 2014 (0.80, IQR: 2.10), and from 2015 to 2019 (1.20, IQR: 3.13). The overall trend of affordability is presented in Figure 2.

Subgroup analysis showed that the cost of medicines of day’s wages in the western region (1.40, IQR: 2.88) was higher than in other regions, which varied largely from the highest in Beijing province (3.04, IQR: 3.24) to the lowest in Shanxi province (0.24, IQR: 0.92). The affordability of urban area (0.95, IQR: 2.80), private sector (0.90, IQR: 2.30), tertiary and secondary health facilities (3.45, IQR: 20.39), and OB drugs (2.90, IQR: 6.68) was higher than their counterparts. The antineoplastic and immunomodulating agents (L) with 5.68 days’ wages required (IQR: 56.47) were higher than other categories of medicines. The results of subgroup analysis of affordability are presented in Figure 3B, among which the provincial characteristics are shown in Figures 4D–F. The detailed results are listed in Supplementary Appendix SA8.

## 4 Discussion

To our knowledge, this is the first systematic review that evaluated the overall price and affordability of essential medicines, their time trends, regional distribution, and health system distribution in China for the last decade. It shows that the price of essential medicines in China is close to and a little higher than the international level, with a tendency to rise first and then fall from 2006 to 2019. In addition, essential medicines are generally affordable in China, though the drug prices compared to the lowest daily wages of government workers increased gradually from 2006 to 2019.

Since 2009, the Chinese government has issued a series of healthcare system reforms aimed to provide equitable basic healthcare for all people and achieve universal health coverage, including the national essential medicine policy. As one significant component of the healthcare system reforms in China, the national essential medicine system had comprehensive and deepen reforms in terms of selection, production, distribution and procurement, reasonable pricing, quality assurance, and evaluation policies, to facilitate the accessibility of essential medicines with the concerted efforts (Tao et al., 2020).

With the persistently high prices than international reference, it is noteworthy that the affordability of essential medicines had modestly risen to more than 1 after 2015, which suggested that the growth rate of medicine price is faster than that of *per capita* income in China in the last decades, and the affordability potentially get worse in the future if without any measures. There are complex factors and issues that trigger the change in medicine prices, such as drug rebates, reimbursement ratio, price deviation and inflation (Long et al., 2022). Establishing a reasonable pricing mechanism for essential medicines is one of the approaches to curb the deterioration, such as reference pricing policy applied in many countries (Acosta et al., 2014). The findings remind us that continued efforts are needed to keep the balance between supply and demand of medicines, and coordinate the price relations to avoid larger deviation from international reference and maintain affordable prices.

The results of subgroup analysis showed that prices of essential medicines in the western region were higher and their affordability (as times of lowest wage per day) was inferior to other regions. Affected by many factors such as historical, social, and natural conditions, its economic development is at the lowest level in China, which is significantly correlated with the shortage of health resources in the western region (Li et al., 2021). The lower economic development level also contributes to the lower residents’ income, and their ability to pay for medications is poorer than that of the developed coastal eastern and central regions (Wang et al., 2022). On the other hand, the western region where far from the coast has more complex geographical conditions and increases the transportation costs for the supply side, which may result in the high price of medicines for this region as well. Therefore, tailored financing policies for medicines, such as relaxing reimbursement, reducing copayment or tiered fixed co-payment of essential medicines may be further needed to lower the out-of-pocket payment of medicines and improve their affordability in those regions (Green et al., 2010; Luiza et al., 2015).

Despite the higher price of essential medicines can be observed in the urban than the rural area, the overall gap of affordability was not significantly widening between urban and rural areas. This might be because residents in urban areas had higher incomes than those who lived in rural areas, allowing them to afford more expensive medicines. On the other hand, the medical insurance system in China plays an important role in reducing people’s burden of using essential medicines, such as the new cooperative medical scheme (NCMS) for rural residents. The reimbursement ratio for medical expenses is more generous in rural areas, and it controls the costs within their affordable ability, which narrow the disparity with the urban area (Xie et al., 2018; Luo et al., 2021).

Furthermore, we found that the public sector provided more affordable prices for essential medicines. Similar findings have been reported in several studies (Guan et al., 2013; Guan et al., 2018; Guan et al., 2018), and it appeared that the private sector also had higher pricing than the public sectors in other low- and middle-income countries (LMICs).(M. Ewen et al., 2017). Previously, the Chinese government allowed public sectors to have a 15% profit margin on medicines, which introduced incentives of over-prescribed, especially expensive medicines (The Central People’s Government of the People’s Republic of China, 2009). Recognizing the caveats, China has implemented two important pharmaceutical policies, namely, zero markup drug policy and centralized drug procurement policy for public health institutions, which have successfully changed the prescribing practice and curbed the rate of increasing expenditure of medicines in China (Liu et al., 2021a; Yuan et al., 2021a). These became significant policy-related factors associated with affordable medicines in public sectors. As national data reported, the growth rate of medicine expenditure has been slowing down in 2019, with an average annual growth of 4.20% lower than that in 2015 (9.89%) (Li et al., 2022). The decreased proportion of medicine costs for patients reduced their financial stress, and previous studies also showed that the incidence of catastrophic health expenditure in China in recent years displayed a downward trend (Liu et al., 2021b; Yuan et al., 2021b).

Additionally, generic medicines presented better affordable than originator products in our review, which was similar to the level among upper-middle-income countries as reported in a previous study (M. Ewen et al., 2017 ). Affordable pricing for medicines has long been a controversial topic, especially for OB. High profits generated by innovative originator products and patent markets for pharmaceutical manufacturers are the primary reason behind the high prices, which makes counterproductive effects on access to these life-saving medicines (Gronde et al., 2017). On the other hand, generic medicine can be purchased at a low price in the market as its low risk in research and development, accordingly protecting equitable opportunities for patients. In order to further improve the quality and bioequivalent recognition, the National Medical Products Administration (NMPA) of China has issued consistency assessment policy for quality and efficacy of generic medicines in 2016 (State Council of the People’s Republic of China, 2016), which promotes generic substitution and assists to decrease medicine expenditure. Further work could be focused on the scope extension of policy implementation to improve the affordability of more medicines.

Our review also indicated that the prices and affordability of essential medicines varied substantially between different ATC categories. It can be seen from the data that although the blood and blood-forming organs drugs (B), antiparasitic products, insecticides and repellents (P), and musculoskeletal system drugs (M) were the top three categories with the highest prices in China compared with the international reference, the affordability of most medicines was less than or close to the daily wage of the lowest paid unskilled government worker, except for antineoplastic and immunomodulating agents (L). The price of most anti-cancer medicines is still in a high position due to the irreplaceability of innovative originator products. Another possible reason for this is the lengthy period of chemotherapy required in cancer patients, which together leads to high total medicine costs. A similar result was reported in a previous study that the anti-cancer medicines with less affordability in LMICs, and it considered that might be associated with the high medicine cost, limited insurance coverage, or non-inclusion in the national essential medicine list (Ocran Mattila et al., 2021). Besides, current systematic reviews indicated that the affordability of essential medicines for asthma, chronic obstructive pulmonary disease, and cardiovascular disease was poor in LMICs, however, which was acceptable in China in our analysis (Babar et al., 2013; Husain et al., 2020; M. Stolbrink et al., 2022). In recent years, China has carried out several rounds of the national-level pricing negotiations and gradually expand the coverage of national health insurance, assisting in the improving accessibility of most medicines, and the patients who required these innovative anti-cancer medicines also got the benefit from the policy (Sun et al., 2022).

This study has several limitations. Firstly, there is insufficient data on the affordability of essential medicine available in China. The coverage of investigation on essential medicines currently performed unbalanced that only 18 provinces (18/31, 58.06%) provided specific data. Further studies are warranted for some provinces where the data is lacking, such as Tibet, Guizhou, Guangxi, Jiangxi, Hainan, Hebei, and Tianjin. Besides, the number of survey studies varied among different investigated provinces and information from individual studies was used to represent the whole group, which may introduce bias in the overall estimates. Moreover, the medicine lists surveyed differed among included studies, which may introduce further heterogeneity of included studies. Unified investigation methods and essential medicine lists of investigation are needed for consistent and long-term monitoring studies in China.

In conclusion, essential medicines were generally affordable in China but remained concerning in the western region, OB drugs, and anti-cancer medicines. This emphasizes the need for concerted national efforts to reduce the disparity of affordability of essential medicines among geographic regions and ATC categories. Evidence-based pricing and purchasing strategies can be exercised to address these issues and improve equitable access to affordable essential medicines in China toward universal health coverage.

## Data Availability

The original contributions presented in the study are included in the article/Supplementary Material, further inquiries can be directed to the corresponding author.
