# Supplementary material for: The price and affordability of essential medicines, progress and regional distribution in China: a systematic review
Source: Front Pharmacol. 2023 May 5;14:1153972. doi: 10.3389/fphar.2023.1153972 (PMC10195994; doi:10.3389/fphar.2023.1153972)
Supplement: Supplementary file 1 [file Table1.DOCX]

***Supplementary Material***

**The price and affordability of essential medicines, progress and regional distribution in China: a systematic review**

**Zheng Liu, Kun Zou, Dan Liu, Miao Zhang, Yuqing Shi, Zhe Chen, Bingchen Lang, Xiao Cheng, Hailong Li, Linan Zeng, Yong Tang, Shaoyang Zhao, Imti Choonara, Yongmu Jiang, Lingli Zhang**

*** Correspondence:** Lingli Zhang, E-mail: zhanglingli@scu.edu.cn

**Contents of Supplementary Materials**

Appendix 1. The detailed search strategies for each database, register, and website

Appendix 2. The region division in China

Appendix 3. List of excluded studies and their excluded reason

Appendix 4. Characteristics of the included studies

Appendix 5. The number of reported studies of each subgroup (n, %)

Appendix 6. The risk of bias assessment of included studies

Appendix 7. The median MPRs of essential medicines in China [Median (IQR)]

Appendix 8. The median affordability of essential medicines in China [Median (IQR)]

Appendix 9. PRISMA Checklist

Appendix 10. Reference list for included studies

**Appendix 1. The detailed search strategies for each database, register, and website**

| **Database** | **Search strategy** | **Result** |
| --- | --- | --- |
| PubMed | ("Drugs, Essential"[Mesh] OR "essential drug*"[Title/Abstract] OR "essential medicine*"[Title/Abstract] OR "essential medication*"[Title/Abstract]) AND ("China"[Title/Abstract] OR "Chinese"[Title/Abstract]) | 204 |
| EMBASE (Ovid) | (("essential drug*" or "essential medicine*" or "essential medication*").ab,kw,ti. or exp essential drug/) AND ("China" or "Chinese").ab,kw,ti. | 345 |
| CENTRAL(Ovid) | (("essential drug*" or "essential medicine*" or "essential medication*").ab,kw,ti. or exp Drugs, Essential/) AND ("China" or "Chinese"). ab,kw,ti. | 12 |
| Web of Science(WOS) | TS=("essential drug*" OR "essential medicine*" OR "essential medication*") AND ("China" OR "Chinese") | 217 |
| The Cochrane Library | (("essential drug*" or "essential medicine*" or "essential medication*"). ab,kw,ti. or exp Drugs, Essential/) AND ("China" or "Chinese"). ab,kw,ti. | 2 |
| China National Knowledge Infrastructure (CNKI) | TI = "essential medicine*" + "essential drug*" | 3351 |
| Chinese BioMedical Literature Database (CBM) | "essential medicine*"[Title] OR " essential drug*"[Title] | 2804 |
| World Health Organization (WHO) | "essential medicine" AND "China" | 3360 |
| International Pharmaceutical Federation (FIP) | essential medicine China | 40 |
| Health Action International (HAL) | essential medicine China | 6 |
| **Total** | | 10341 |

**Appendix 2. The region division in China**

| **Region** | **Province** |
| --- | --- |
| Eastern | Beijing, Fujian, Guangdong, Hainan, Hebei, Jiangsu, Shandong, Shanghai, Tianjin, Zhejiang |
| Central | Anhui, Henan, Hubei, Hunan, Jiangxi, Shanxi |
| Western | Chongqing, Gansu, Guangxi, Guizhou, Inner Mongolia, Ningxia, Qinghai, Shaanxi, Sichuan, Tibet, Xinjiang, Yunnan |
| Northeastern | Heilongjiang, Jilin, Liaoning |

**Appendix 3. List of excluded studies and their excluded reason**

| **Number** | **Author, year** | **Title** | **Reason for exclusion** |
| --- | --- | --- | --- |
| 1 | Su 2017 | Availability, cost, and prescription patterns of antihypertensive medications in primary health care in China: a nationwide cross-sectional survey | Improper formula of outcome: using the annual cost of medicine rather than retail price per sale unit |
| 2 | Fang 2013 | Access to affordable medicines after health reform: Evidence from two cross-sectional surveys in Shaanxi Province, western China | Improper formula of outcome: using the procurement prices rather than the retail price |
| 3 | Wang 2015 | Short-term differences in drug prices after implementation of the national essential medicines system: A case study in rural Jiangxi Province, China | Improper formula of outcome: using the lowest retail prices rather than the median price |
| 4 | Dai 2013 | Effect on implementing essential medicine system at primary healthcare institutions in Henan Province [Chinese] | Improper formula of outcome: using the mean retail prices rather than the median price |
| 5 | Wang 2014 | Comparative study on the price and profit rate of essential medicines in rural areas [Chinese] | Improper formula of outcome: using the mean retail prices rather than the median price |
| 6 | Wang(1) 2013 | Measures and advices for sale prices of essential drugs in primary medical institution in Jiangxi [Chinese] | Improper formula of outcome: using the mean retail prices rather than the median price |
| 7 | Guo 2012 | Comparative analysis on the price of essential medicines: Based on the sample survey of primary health care institutions and pharmacies in 18 provinces [Chinese] | Improper formula of outcome: using the mean retail prices rather than the median price |
| 8 | He 2011 | Comparative analysis on the drug price of township health center before and after the essential medicines system reform：Based on the sample survey of three counties in Anhui province [Chinese] | Unclear data: unclear to the attributes of prices |
| 9 | Chen 2016 | The difference analysis of essential drugs' prices on township health center and retail drug stores in Guizhou [Chinese] | Improper formula of outcome: using the mean retail prices rather than the median price |
| 10 | Chen 2012 | Survey and analysis of retail prices of essential medicines in the rural areas of Jiangxi [Chinese] | Improper formula of outcome: using the ratio of the mean retail prices to national guiding price |
| 11 | He 2016 | Analysis on centralized purchasing price of twenty kinds of essential drugs [Chinese] | Improper formula of outcome: using the procurement prices and bidding price rather than the retail price |
| 12 | Yang(1) 212 | Analysis on the bidding drug varieties in the centralized bidding of essential drug in Sichuan province in 2011 [Chinese] | Improper formula of outcome: using the bidding price rather than the retail price |
| 13 | Yang 2015 | Study on the influence mechanism of essential medicine centralized purchasing policy on medicine price in Chongqing [Chinese] | Improper formula of outcome: using the procurement prices rather than the retail price |
| 14 | Zhang 2011 | Impact of essential drugs system on economic burden of rural residents during implement of the new rural cooperative medical scheme [Chinese] | Improper formula of outcome: using the procurement prices rather than the retail price |
| 15 | Xin 2012 | Comparative analysis of procurement prices of essential medicines in China [Chinese] | Improper formula of outcome: using the procurement prices rather than the retail price |
| 16 | Wang 2015 | Study on the price components of pediatric essential medicines in Shaanxi province [Chinese] | Inconsistent outcome: price components |
| 17 | Fang 2017 | The evaluation study of the fairness of national essential medicine system in Anhui province [Chinese] | Inconsistent outcome: price index |
| 18 | He 2021 | Study on the allocation, use and price of essential medicines: based on data analysis of primary health institutions in Hubei province from 2015 to 2018 [Chinese] | Inconsistent outcome: price index |
| 19 | Ma 2015 | Price comparisons of the essential and non-essential antimicrobial drugs among national reimbursement drug list in Tianjin, China [Chinese] | Inconsistent outcome: price index |
| 20 | Song 2013 | Analysis on changes of drug price after implementing of the essential drug system in primary health care institutions: based on the empirical study of the four provinces (autonomous regions) in China [Chinese] | Inconsistent outcome: price index |
| 21 | Zhu 2020 | Study on the trend of availability and price of the low-cost drugs [Chinese] | Inconsistent outcome: price index |
| 22 | Liu 2020 | Study on the price level of essential drugs based on price index: take anti-infective drugs in Jiangsu province as an example [Chinese] | Inconsistent outcome: price index |
| 23 | Ma 2014 | Theoretical and empirical study on the drug price level: based on the basic medical insurance drugs in Tianjin, China [Chinese] | Inconsistent outcome: price index |
| 24 | Yang 2019 | Research on the retail market price of essential drugs in Changchun [Chinese] | Lacking data |
| 25 | Li(1) 2011 | Study on policy effects of essential medicines system in rural areas of Shandong province: evidence from two pilot counties [Chinese] | Inconsistent outcome: drug cost per case |
| 26 | Wang(2) 2013 | Research on implementation effect of essential medicine policy in county level hospital from the perspective of sustainability theory: take Anhui province for example [Chinese] | Lacking data |
| 27 | Lin 2012 | Evaluation of the implementation of essential medicines system in Fujian primary care health institutions: took Fuzhou and Xiamen as examples [Chinese] | Inconsistent outcome: average cost per prescription and drug price ratio |
| 28 | Guo 2020 | Study on the effects of full coverage policy for essential antihypertensive medicines on the equity of medicine expenditure affordability in Taizhou city [Chinese] | Inconsistent outcome: catastrophic expenditure on medicines |
| 29 | Zhang 2019 | Analysis of the impacts of the full coverage policy for essential medicines on the fairness of medicine expenditure affordability of diabetic patients in Taizhou city [Chinese] | Inconsistent outcome: catastrophic expenditure on medicines |
| 30 | Li(2) 2011 | Analysis of the utilization and price of national essential drugs in our hospital [Chinese] | Improper formula of outcome: using the procurement prices rather than the retail price |
| 31 | Yang(2) 2012 | Analysis on the bidding drug prices and its sale-permission in the centralized bidding of essential drug in Sichuan province in 2011 [Chinese] | Lacking data |
| 32 | Fang 2016 | Studying on the calculation methods and strategies on the affordability of essential medicines in China [Chinese] | Review |
| 33 | Li 2014 | Research on implementation of national essential medicine system in Beijing [Chinese] | Review |
| 34 | Wang 2015 | Analysis of current situation and countermeasure of essential medicine price management in China [Chinese] | Review |
| 35 | Wang 2012 | Effect evaluation of national essential medicine system in China: based on survey data from Shandong, Hubei and Sichuan [Chinese] | Review |
| 36 | Zhang 2012 | Discussion of empirical study on accessibility of essential drug in China [Chinese] | Review |

**Appendix 4. Characteristics of the included studies**

| Author, year | Survey year | Region | Province | Area | Number of health facilities | Types of health facilities | Levels of health facilities | Methodology | Report outcome |
| --- | --- | --- | --- | --- | --- | --- | --- | --- | --- |
| Wang 2021 | 2016, 2018 | eastern | Jiangsu | urban | 56 | public, private | tertiary, secondary, primary | WHO/HAI | MPR, affordability |
| Li 2019 | 2016 | western | Shaanxi | urban, rural | 21 | public, private | tertiary, secondary | WHO/HAI | MPR, affordability |
| Wang 2017 | 2014 | nationwide | NR | urban, rural | NR | public, private | NR | WHO/HAI | MPR, affordability |
| Jiang 2015 | 2012 | western | Shaanxi | urban, rural | 240 | public, private | tertiary, secondary, primary | WHO/HAI | MPR, affordability |
| Wang(1) 2014 | 2012 | western | Shaanxi | urban, rural | 60 | public, private | tertiary, secondary, primary | WHO/HAI | MPR |
| Yang 2010 | 2007 | central | Hubei | urban, rural | 36 | public, private | tertiary, secondary, primary | WHO/HAI | MPR, affordability |
| Xu 2020 | 2015 | central | Anhui | rural | 143 | public | primary | WHO/HAI | MPR, affordability |
| Yang 2019 | 2015 | western | Shaanxi | urban, rural | 144 | public, private | tertiary, secondary, primary | WHO/HAI | MPR, affordability |
| Guan 2018 | 2011-2016 | nationwide | NR | urban, rural | 1159 | public | tertiary, secondary | WHO/HAI | MPR |
| Yang 2020 | 2010, 2012, 2014, 2018 | nationwide | Shaanxi; Shandong; Hubei; Henan; Yunnan | urban, rural | 519 | public, private | tertiary, secondary, primary | WHO/HAI | MPR |
| Chen 2021 | 2019 | central | Hubei | urban | 28 | public | tertiary, secondary | WHO/HAI | MPR, affordability |
| Dong 2020 | 2018 | eastern | Zhejiang | urban, rural | 60 | public, private | tertiary, secondary, primary | WHO/HAI | MPR, affordability |
| Gong 2018 | 2016 | central | Hubei | urban, rural | 34 | public | tertiary, secondary, primary | WHO/HAI | affordability |
| Zhu 2019 | 2012, 2016 | eastern | Jiangsu | urban, rural | 60 | public | tertiary, secondary | WHO/HAI | affordability |
| Song 2018 | 2009, 2010 | nationwide | Shandong; Zhejiang; Anhui; Ningxia | urban | 146 | public | primary | WHO/HAI | MPR, affordability |
| Liu 2017 | 2016 | central | Hubei | urban | 60 | public, private | tertiary, secondary, primary | WHO/HAI | MPR, affordability |
| Xi 2015 | 2013 | eastern | Jiangsu | urban, rural | 63 | public, private | tertiary, secondary, primary | WHO/HAI | affordability |
| Zhu 2021 | 2013-2019 | eastern | Jiangsu | urban, rural | 41 | public | tertiary, secondary, primary | WHO/HAI | affordability |
| Sun 2018 | 2017 | eastern | Jiangsu | urban, rural | 60 | public, private | tertiary, secondary, primary | WHO/HAI | MPR, affordability |
| Wu 2018 | 2016 | central | Hubei | urban | 33 | public | tertiary, secondary, primary | WHO/HAI | MPR, affordability |
| Yang 2016 | 2015 | northeastern | Jilin | urban | 146 | public, private | tertiary, secondary, primary | WHO/HAI | MPR |
| Chen 2019 | 2015-2017 | central | Hubei | urban, rural | NR | public | primary | WHO/HAI | affordability |
| Chen 2011 | 2009, 2010 | eastern | Fujian | urban, rural | 193 | public | primary | WHO/HAI | MPR |
| Dai 2013 | 2009, 2011 | central, western | Anhui; Henan; Chongqing | rural | 30 | public | primary | other | MPR |
| Dai 2020 | 2017 | nationwide | NR | urban | 55 | public | tertiary, secondary, primary | WHO/HAI | MPR, affordability |
| Dou 2017 | 2015 | central | Henan | urban | 5 | public | secondary, primary | WHO/HAI | affordability |
| Guan(1) 2013 | 2010 | nationwide | NR | urban, rural | 347 | public, private | tertiary, secondary, primary | WHO/HAI | MPR |
| Guan(2) 2013 | 2010 | nationwide | NR | urban, rural | 347 | public, private | tertiary, primary | WHO/HAI | affordability |
| Jiang(1) 2013 | 2012 | western | Shaanxi | urban, rural | 120 | public, private | tertiary, secondary, primary | WHO/HAI | MPR, affordability |
| Jiang(2) 2013 | 2010, 2012 | western | Shaanxi | urban, rural | 108 | private | tertiary, secondary, primary | WHO/HAI | MPR, affordability |
| Jiang 2019 | 2017 | northeastern | Liaoning | urban, rural | 62 | public | tertiary, secondary, primary | WHO/HAI | MPR, affordability |
| Jiang 2017 | 2014 | western | Shaanxi | urban, rural | 10 | public, private | tertiary, secondary, primary | WHO/HAI | MPR, affordability |
| Li(1) 2011 | 2010 | eastern | Guangdong | urban | 29 | public | tertiary, secondary, primary | WHO/HAI | affordability |
| Li(1) 2013 | 2010, 2011 | eastern | Beijing | urban | 1 | public | tertiary | other | affordability |
| Li 2009 | 2008 | central | Hubei | rural | 36 | public, private | primary | WHO/HAI | MPR, affordability |
| Zhang(1) 2015 | 2013 | eastern | Jiangsu | urban | 60 | public, private | tertiary, primary | WHO/HAI | affordability |
| Zhang(1) 2013 | 2012 | western | Qinghai; Ningxia; Gansu; Xinjiang; Inner Mongolia | urban | 84 | public, private | tertiary, secondary, primary | WHO/HAI | affordability |
| Zhang(2) 2015 | 2012, 2013 | western | Sichuan | urban | 1 | public | tertiary | other | affordability |
| Li 2012 | 2011 | eastern | Shanghai | urban, rural | 6 | public | primary | WHO/HAI | affordability |
| Li(2) 2013 | 2011 | eastern | Zhejiang | urban | 17 | public, private | primary | WHO/HAI | affordability |
| Lin 2013 | 2009, 2010 | western | Sichuan | rural | 168 | public | primary | WHO/HAI | MPR, affordability |
| Ma 2017 | 2016 | eastern | Jiangsu | urban, rural | NR | public | primary | WHO/HAI | affordability |
| Shan 2016 | 2013 | eastern | Beijing | urban, rural | NR | public | tertiary, secondary, primary | WHO/HAI | MPR |
| Shen 2018 | 2015 | eastern | Shanghai | urban | NR | NR | NR | WHO/HAI | affordability |
| Shen 2017 | 2014 | eastern | Shanghai | urban | NR | NR | NR | WHO/HAI | MPR |
| Song 2012 | 2009, 2010 | nationwide | Anhui; Zhejiang; Shandong; Ningxia | rural | 150 | public | primary | WHO/HAI | MPR, affordability |
| Tan 2014 | 2009, 2011 | central | Shanxi | urban, rural | NR | public | primary | WHO/HAI | MPR, affordability |
| Wang 2011(1) | 2010 | nationwide | Beijing; Shanxi; Liaoning; Jilin; Heilongjiang; Fujian; Henan; Hubei; Hunan; Sichuan; Xinjiang | urban | 50 | public, private | tertiary, secondary, primary | WHO/HAI | MPR |
| Wang(2) 2014 | 2011 | western | Shaanxi | urban | 30 | private | primary | WHO/HAI | MPR |
| Wang(3) 2014 | 2012 | western | Shaanxi | urban | 60 | public | tertiary, secondary, primary | WHO/HAI | MPR |
| Wang 2020 | 2019 | eastern | Shandong | urban, rural | 71 | public, private | tertiary, secondary, primary | WHO/HAI | MPR |
| Wei 2019 | 2012, 2016 | eastern | NR | urban, rural | 1725 | public | tertiary, secondary, primary | WHO/HAI | MPR, affordability |
| Wei 2013 | 2009, 2011 | eastern | Shandong | rural | 18 | public | primary | WHO/HAI | MPR, affordability |
| Xu(1) 2012 | 2011 | nationwide | NR | urban, rural | NR | public | tertiary, secondary, primary | WHO/HAI | affordability |
| Xu(2) 2012 | 2010 | eastern | Jiangsu | urban, rural | NR | public, private | tertiary, secondary, primary | WHO/HAI | affordability |
| Yang 2009 | 2007 | central | Hubei | rural | 36 | public, private | primary | WHO/HAI | MPR, affordability |
| Yang 2012 | 2010 | eastern | Shandong | rural | 9 | public | primary | WHO/HAI | MPR, affordability |
| Li(2) 2011 | 2010 | eastern | Guangdong; | urban | 28 | public | tertiary, secondary, primary | WHO/HAI | MPR, affordability |
| Wang 2011(2) | 2010 | nationwide | Beijing; Shanxi; Liaoning; Jilin; Heilongjiang; Fujian; Henan; Hubei; Hunan; Sichuan; Xinjiang | urban, rural | 50 | public, private | tertiary, secondary, primary | WHO/HAI | affordability |
| Yang 2008 | 2007 | central | Hubei | rural | 7 | public, private | primary | WHO/HAI | affordability |
| Ye 2008 | 2006 | eastern | Shanghai | urban | 50 | public, private | tertiary, secondary, primary | WHO/HAI | affordability |
| Yin 2013 | 2011 | western | Ningxia | urban | 1 | public | primary | WHO/HAI | MPR, affordability |
| Zhang 2020 | 2019 | eastern | Jiangsu | urban | 13 | public | tertiary, secondary | WHO/HAI | MPR, affordability |
| Zhang 2012 | 2009, 2010 | eastern | Fujian | urban, rural | 189 | public | tertiary, secondary, primary | WHO/HAI | MPR |
| Zhang(2) 2013 | 2010 | nationwide | NR | urban, rural | 38 | public | primary | other | MPR |

NR: Not report; WHO/ HAI: The World Health Organization/ Health Action International;

**Appendix 5. The number of reported studies of each subgroup (n, %)**

| Period | | MPRs | | | |  | affordability | | | |
| --- | --- | --- | --- | --- | --- | --- | --- | --- | --- | --- |
|  |  | Before 2009 | 2010-2014 | 2015-2019 | Overall |  | Before 2009 | 2010-2014 | 2015-2019 | Overall |
| Overall | | 11 (25.00) | 26 (59.09) | 17 (38.64) | 44 (100.00) |  | 10 (20.00) | 28 (56.00) | 20 (40.00) | 50 (100.00) |
| Region | Eastern | 3 (6.82) | 11 (25.00) | 8 (18.18) | 17 (38.64) |  | 2 (4.00) | 13 (26.00) | 9 (18.00) | 20 (40.00) |
|  | Central | 5 (11.36) | 5 (11.36) | 6 (13.64) | 13 (29.55) |  | 5 (10.00) | 1 (2.00) | 7 (14.00) | 12 (24.00) |
|  | Western | 2 (4.55) | 13 (29.55) | 4 (9.09) | 16 (36.36) |  | 1 (2.00) | 8 (16.00) | 2 (4.00) | 10 (20.00) |
|  | Northeastern | / | / | 2 (4.55) | 2 (4.55) |  | / | / | 1 (2.00) | 1 (2.00) |
| Province | Anhui | 1 (2.27) | 1 (2.27) | 1 (2.27) | 2 (4.55) |  | / | / | 1 (2.00) | 1 (2.00) |
|  | Beijing | / | 1 (2.27) | / | 1 (2.27) |  | / | 1 (2.00) | / | 1 (2.00) |
|  | Chongqing | 1 (2.27) | 1 (2.27) | / | 1 (2.27) |  | / | / | / | / |
|  | Fujian | 2 (4.55) | 2 (4.55) | / | 2 (4.55) |  | / | / | / | / |
|  | Gansu | / | / | / | / |  | / | / | / | / |
|  | Guangdong | / | 1 (2.27) | / | 1 (2.27) |  | / | 2 (4.00) | / | 2 (4.00) |
|  | Guangxi | / | / | / | / |  | / | / | / | / |
|  | Guizhou | / | / | / | / |  | / | / | / | / |
|  | Hainan | / | / | / | / |  | / | / | / | / |
|  | Hebei | / | / | / | / |  | / | / | / | / |
|  | Henan | 1 (2.27) | 1 (2.27) | 1 (2.27) | 2 (4.55) |  | / | / | 1 (2.00) | 1 (2.00) |
|  | Heilongjiang | / | / | / | / |  | / | / | / | / |
|  | Hubei | 3 (6.82) | / | 4 (9.09) | 7 (15.91) |  | 4 (8.00) | / | 5 (10.00) | 9 (18.00) |
|  | Hunan | / | / | / | / |  | / | / | / | / |
|  | Inner Mongolia | / | / | / | / |  | / | / | / | / |
|  | Jiangsu | / | / | 3 (6.82) | 3 (6.82) |  | / | 5 (10.00) | 6 (12.00) | 9 (18.00) |
|  | Jiangxi | / | / | / | / |  | / | / | / | / |
|  | Jilin | / | / | 1 (2.27) | 1 (2.27) |  | / | / | / | / |
|  | Liaoning | / | / | 1 (2.27) | 1 (2.27) |  | / | / | 1 (2.00) | 1 (2.00) |
|  | Ningxia | / | 1 (2.27) | / | 1 (2.27) |  | / | 1 (2.00) | / | 1 (0.00) |
|  | Qinghai | / | / | / | / |  | / | / | / | / |
|  | Shaanxi | / | 7 (15.91) | 3 (6.82) | 10 (22.73) |  | / | 4 (8.00) | 2 (4.00) | 6 (12.00) |
|  | Shandong | 1 (2.27) | 2 (4.55) | 2 (4.55) | 4 (9.09) |  | 1 (2.00) | 2 (4.00) | / | 2 (4.00) |
|  | Shanghai | / | 1 (2.27) | / | 1 (2.27) |  | 1 (2.00) | 1 (2.00) | 1 (2.00) | 3 (6.00) |
|  | Shanxi | 1 (2.27) | 1 (2.27) | / | 1 (2.27) |  | 1 (2.00) | 1 (2.00) | / | 1 (2.00) |
|  | Sichuan | 1 (2.27) | 1 (2.27) | / | 1 (2.27) |  | 1 (2.00) | 2 (4.00) | / | 2 (4.00) |
|  | Tianjin | / | / | / | / |  | / | / | / | / |
|  | Tibet | / | / | / | / |  | / | / | / | / |
|  | Xinjiang | / | / | / | / |  | / | / | / | / |
|  | Yunnan | / | / | 1 (2.27) | 1 (2.27) |  | / | / | / | / |
|  | Zhejiang | / | / | 1 (2.27) | 1 (2.27) |  | / | 1 (2.00) | 1 (2.00) | 2 (4.00) |
| Area | Urban | 1 (2.27) | 7 (15.91) | 7 (15.91) | 14 (31.82) |  | 3 (6.00) | 14 (28.00) | 13 (26.00) | 26 (52.00) |
|  | Rural | 6 (13.64) | 5 (11.36) | 1 (2.27) | 8 (18.18) |  | 7 (14.00) | 9 (18.00) | 6 (12.00) | 17 (34.00) |
| Types of health facilities | Public | 11 (25.00) | 21 (47.73) | 16 (36.36) | 38 (86.36) |  | 10 (20.00) | 25 (50.00) | 19 (38.00) | 46 (92.00) |
|  | Private | 2 (4.55) | 7 (15.91) | 8 (18.18) | 17 (38.64) |  | 5 (10.00) | 10 (20.00) | 5 (10.00) | 20 (40.00) |
| Levels of health facilities | Primary | 9 (20.45) | 11 (25.00) | 4 (9.09) | 17 (38.64) |  | 8 (16.00) | 14 (28.00) | 4 (8.00) | 21 (42.00) |
|  | Secondary | / | / | 3 (6.82) | 3 (6.82) |  | / | / | 2 (4.00) | 2 (4.00) |
|  | Tertiary | / | / | 3 (6.82) | 3 (6.82) |  | / | 4 (8.00) | 2 (4.00) | 6 (12.00) |
|  | Tertiary & Secondary | / | 1 (2.27) | 4 (9.09) | 4 (9.09) |  | / | 4 (8.00) | 3 (6.00) | 6 (12.00) |
|  | Secondary & Primary | / | / | / | / |  | / | / | 1 (2.00) | 1 (2.00) |
|  | Tertiary & Secondary& Primary | 2 (4.55) | 12 (27.27) | 11 (25.00) | 23 (52.27) |  | 2 (4.00) | 11 (22.00) | 10 (20.00) | 21 (42.00) |
| Characteristic (OB0LPG) | OB | 2 (4.55) | 11 (25.00) | 14 (31.82) | 26 (59.09) |  | 3 (6.00) | 17 (34.00) | 13 (26.00) | 30 (60.00) |
|  | LPG | 1 (2.27) | 10 (22.73) | 14 (31.82) | 24 (54.55) |  | 3 (6.00) | 17 (34.00) | 13 (26.00) | 30 (60.00) |
| ATC | A | 11 (25.00) | 19 (43.18) | 10 (22.73) | 31 (70.45) |  | 10 (20.00) | 23 (46.00) | 12 (24.00) | 39 (78.00) |
|  | B | 3 (6.82) | 9 (20.45) | 5 (11.36) | 13 (29.55) |  | 1 (2.00) | 8 (16.00) | 2 (4.00) | 9 (18.00) |
|  | C | 11 (25.00) | 18 (40.91) | 5 (11.36) | 25 (56.82) |  | 10 (20.00) | 23 (46.00) | 8 (16.00) | 35 (70.00) |
|  | D | 1 (2.27) | 3 (6.82) | 1 (2.27) | 4 (9.09) |  | / | / | 1 (2.00) | 1 (2.00) |
|  | G | / | 1 (2.27) | 1 (2.27) | 2 (4.55) |  | / | / | 1 (2.00) | 1 (2.00) |
|  | H | 3 (6.82) | 6 (13.64) | 5 (11.36) | 10 (22.73) |  | 1 (2.00) | 3 (6.00) | 3 (6.00) | 6 (12.00) |
|  | J | 10 (22.73) | 19 (43.18) | 8 (18.18) | 28 (63.64) |  | 10 (20.00) | 23 (46.00) | 9 (18.00) | 36 (72.00) |
|  | L | / | / | 2 (4.55) | 2 (4.55) |  | / | 1 (2.00) | 3 (6.00) | 3 (6.00) |
|  | M | 11 (25.00) | 17 (38.64) | 4 (9.09) | 23 (52.27) |  | 10 (20.00) | 18 (36.00) | 4 (8.00) | 26 (52.00) |
|  | N | 9 (20.45) | 18 (40.91) | 5 (11.36) | 24 (54.55) |  | 8 (16.00) | 16 (32.00) | 7 (14.00) | 26 (52.00) |
|  | P | 9 (20.45) | 12 (27.27) | 3 (6.82) | 17 (38.64) |  | 4 (8.00) | 5 (10.00) | 1 (2.00) | 5 (10.00) |
|  | R | 6 (13.64) | 12 (27.27) | 5 (11.36) | 19 (43.18) |  | 5 (10.00) | 14 (28.00) | 6 (12.00) | 23 (46.00) |
|  | S | 1 (2.27) | 1 (2.27) | 1 (2.27) | 2 (4.55) |  | / | / | / | / |
|  | V | / | / | 1 (2.27) | 1 (2.27) |  | / | / | 1 (2.00) | 1 (2.00) |
|  | Z | / | 5 (11.36) | 4 (9.09) | 8 (18.18) |  | / | 6 (12.00) | 1 (2.00) | 6 (12.00) |

MPRs: median price ratios; OB: originator brand; LPG: lowest priced generic equivalent; ATC: anatomic therapeutic chemistry; A: alimentary tract and metabolism; B: blood and blood forming organs; C: cardiovascular system; D: dermatologicals; G: genito urinary system and sex hormones; H: systemic hormonal preparations, excl. sex hormones and insulins; J: antiinfectives for systemic use; L: antineoplastic and immunomodulating agents; M: musculoskeletal system; N: nervous system; P: antiparasitic products, insecticides and repellents; R: respiratory system; S: sensory organs; V: various; Z: unknow.

**Appendix 6. The risk of bias assessment of included studies**

| Author, year | 1. Was the sample frame appropriate to address the target population? | 2. Were study participants sampled in an appropriate way? | 3. Was the sample size adequate? | 4. Were the study subjects and the setting described in detail? | 5. Was the data analysis conducted with sufficient coverage of the identified sample? | 6. Were valid methods used for the identification of the condition? | 7. Was the condition measured in a standard, reliable way for all participants? | 8. Was there appropriate statistical analysis? | 9. Was the response rate adequate, and if not, was the low response rate managed appropriately? | Total Score | Quality |
| --- | --- | --- | --- | --- | --- | --- | --- | --- | --- | --- | --- |
| Wang 2021 | Y | Y | Y | Y | Y | Y | Y | Y | U | 8 | high |
| Li 2019 | Y | N | N | Y | Y | Y | Y | Y | Y | 7 | high |
| Wang 2017 | N | N | U | N | U | Y | Y | Y | U | 3 | low |
| Jiang 2015 | Y | N | Y | Y | N | Y | U | Y | U | 5 | moderate |
| Wang(1) 2014 | Y | Y | Y | Y | Y | Y | Y | Y | U | 8 | high |
| Yang 2010 | Y | Y | Y | Y | Y | Y | Y | Y | U | 8 | high |
| Xu 2020 | Y | Y | Y | N | Y | Y | Y | Y | Y | 8 | high |
| Yang 2019 | Y | Y | Y | Y | N | Y | Y | Y | U | 7 | high |
| Guan 2018 | Y | N | U | Y | Y | Y | Y | Y | U | 6 | moderate |
| Yang 2020 | Y | Y | Y | Y | Y | Y | Y | Y | U | 8 | high |
| Chen 2021 | Y | N | N | Y | N | Y | Y | Y | U | 5 | moderate |
| Dong 2020 | Y | Y | Y | Y | N | Y | Y | Y | U | 7 | high |
| Gong 2018 | Y | Y | N | Y | N | Y | N | Y | N | 5 | moderate |
| Zhu 2019 | Y | Y | Y | Y | Y | Y | Y | Y | U | 8 | high |
| Song 2018 | Y | Y | Y | Y | Y | Y | Y | Y | U | 8 | high |
| Liu 2017 | Y | N | N | Y | Y | Y | Y | Y | U | 6 | moderate |
| Xi 2015 | Y | Y | Y | Y | N | Y | Y | Y | U | 7 | high |
| Zhu 2021 | Y | N | Y | Y | N | Y | U | Y | U | 5 | moderate |
| Sun 2018 | Y | N | Y | Y | N | Y | Y | Y | U | 6 | moderate |
| Wu 2018 | Y | U | Y | Y | N | Y | Y | Y | U | 6 | moderate |
| Yang 2016 | Y | Y | Y | Y | Y | Y | Y | Y | U | 8 | high |
| Chen 2019 | Y | N | U | N | N | Y | Y | Y | U | 4 | moderate |
| Chen 2011 | Y | U | U | N | Y | Y | N | Y | U | 4 | moderate |
| Dai 2013 | Y | N | N | N | N | N | N | Y | U | 2 | low |
| Dai 2020 | Y | N | U | N | N | Y | Y | Y | U | 4 | moderate |
| Dou 2017 | Y | N | N | N | Y | Y | N | Y | U | 4 | moderate |
| Guan(1) 2013 | Y | U | Y | Y | Y | Y | Y | Y | Y | 8 | high |
| Guan(2) 2013 | Y | U | Y | Y | Y | Y | Y | Y | Y | 8 | high |
| Jiang(1) 2013 | Y | N | Y | Y | N | Y | Y | Y | U | 6 | moderate |
| Jiang(2) 2013 | Y | N | Y | N | N | Y | Y | Y | U | 5 | moderate |
| Jiang 2019 | Y | Y | N | Y | Y | Y | N | Y | Y | 7 | high |
| Jiang 2017 | Y | N | Y | Y | Y | Y | N | Y | U | 6 | moderate |
| Li(1) 2011 | Y | Y | Y | Y | N | Y | U | Y | U | 6 | moderate |
| Li(1) 2013 | N | N | N | N | Y | N | N | Y | NA | 3 | low |
| Li 2009 | Y | Y | Y | Y | Y | Y | Y | Y | U | 8 | high |
| Zhang(1) 2015 | Y | Y | Y | Y | N | Y | U | Y | Y | 7 | high |
| Zhang(1) 2013 | Y | Y | Y | Y | N | Y | U | U | N | 5 | moderate |
| Zhang(2) 2015 | N | N | N | N | Y | N | N | Y | NA | 3 | low |
| Li 2012 | Y | Y | U | N | Y | Y | N | U | U | 4 | moderate |
| Li(2) 2013 | Y | Y | N | Y | N | Y | U | Y | U | 5 | moderate |
| Lin 2013 | Y | Y | Y | Y | Y | Y | U | Y | Y | 8 | high |
| Ma 2017 | Y | N | N | Y | N | Y | U | Y | U | 4 | moderate |
| Shan 2016 | Y | N | Y | Y | Y | Y | N | U | U | 5 | moderate |
| Shen 2018 | N | N | U | N | Y | Y | N | Y | NA | 4 | moderate |
| Shen 2017 | N | N | U | N | Y | Y | N | Y | NA | 4 | moderate |
| Song 2012 | Y | Y | Y | Y | Y | Y | U | Y | U | 7 | high |
| Tan 2014 | N | N | N | N | Y | Y | U | Y | NA | 4 | moderate |
| Wang 2011(1) | Y | N | N | N | Y | Y | U | Y | N | 4 | moderate |
| Wang(2) 2014 | Y | N | Y | Y | Y | Y | Y | Y | U | 7 | high |
| Wang(3) 2014 | Y | N | Y | Y | Y | Y | Y | Y | U | 7 | high |
| Wang 2020 | Y | N | Y | Y | N | Y | Y | Y | U | 6 | moderate |
| Wei 2019 | Y | N | Y | Y | Y | Y | N | Y | U | 6 | moderate |
| Wei 2013 | Y | Y | N | Y | Y | Y | U | Y | U | 6 | moderate |
| Xu(1) 2012 | N | N | U | N | Y | Y | N | Y | NA | 4 | moderate |
| Xu(2) 2012 | N | N | U | N | Y | Y | N | Y | NA | 4 | moderate |
| Yang 2009 | Y | Y | N | Y | N | Y | N | Y | U | 5 | moderate |
| Yang 2012 | Y | Y | N | Y | Y | Y | Y | Y | U | 7 | high |
| Li(2) 2011 | Y | Y | Y | Y | N | Y | N | Y | U | 6 | moderate |
| Wang 2011(2) | Y | Y | N | N | Y | Y | Y | Y | N | 6 | moderate |
| Yang 2008 | Y | Y | N | Y | Y | Y | U | Y | U | 6 | moderate |
| Ye 2008 | Y | Y | Y | Y | N | Y | Y | Y | U | 7 | high |
| Yin 2013 | N | N | N | N | U | Y | N | Y | NA | 3 | low |
| Zhang 2020 | Y | Y | Y | Y | N | Y | U | Y | U | 6 | moderate |
| Zhang 2012 | Y | Y | Y | Y | Y | Y | U | Y | U | 7 | high |
| Zhang(2) 2013 | Y | N | U | Y | N | N | N | Y | NA | 4 | moderate |

Y: Yes; N: No; U: Unclear; NA: Not applicable

**Appendix 7. The median MPRs of essential medicines in China [Median (IQR)]**

| Period | | Before 2009 | 2010-2014 | 2015-2019 | Overall |
| --- | --- | --- | --- | --- | --- |
| Overall | | 1.15 (2.40) | 1.86 (7.65) | 1.51 (4.01) | 1.59 (5.39) |
| Region | Eastern | 0.98 (2.39) | 1.34 (7.31) | 1.38 (4.72) | 1.22 (5.75) |
|  | Central | 1.14 (1.88) | 2.40 (6.99) | 1.04 (1.02) | 1.20 (2.44) |
|  | Western | 1.65 (4.02) | 2.27 (8.50) | 4.66 (9.68) | **2.50 (8.29)** |
|  | Northeastern | / | / | 2.10 (4.46) | 2.10 (4.46) |
| Province | Anhui | 1.32 (3.31) | 0.70 (0.81) | 1.12 (3.14) | 1.10 (3.05) |
|  | Beijing | / | 8.00 (13.90) | / | 8.00 (13.90) |
|  | Chongqing | 0.93 (6.45) | 1.13 (3.64) | / | 1.04 (5.04) |
|  | Fujian | 0.92 (1.97) | 0.65 (1.11) | / | 0.77 (1.28) |
|  | Gansu | / | / | / | / |
|  | Guangdong | / | 0.77 (1.91) | / | 0.77 (1.91) |
|  | Guangxi | / | / | / | / |
|  | Guizhou | / | / | / | / |
|  | Hainan | / | / | / | / |
|  | Hebei | / | / | / | / |
|  | Henan | 2.36 (6.34) | 1.19 (3.86) | 6.14 (11.00) | 1.98 (5.40) |
|  | Heilongjiang | / | / | / | / |
|  | Hubei | 1.02 (1.70) | / | 1.02 (0.61) | 1.02 (1.00) |
|  | Hunan | / | / | / | / |
|  | Inner Mongolia | / | / | / | / |
|  | Jiangsu | / | / | 0.71 (2.52) | 0.71 (2.52) |
|  | Jiangxi | / | / | / | / |
|  | Jilin | / | / | 5.10 (5.76) | 5.10 (5.76) |
|  | Liaoning | / | / | 1.53 (4.08) | 1.53 (4.08) |
|  | Ningxia | / | 2.54 (5.81) | / | 2.54 (5.81) |
|  | Qinghai | / | / | / | / |
|  | Shaanxi | / | 2.23 (8.68) | 4.66 (11.03) | 2.61 (8.77) |
|  | Shandong | 1.76 (5.93) | 0.79 (2.03) | 3.85 (9.14) | 1.71 (6.34) |
|  | Shanghai | / | 7.48 (13.23) | / | 7.48 (13.23) |
|  | Shanxi | 2.16 (8.50) | 2.02 (5.31) | / | 2.14 (5.31) |
|  | Sichuan | 1.74 (3.52) | 0.66 (0.90) | / | 1.13 (2.28) |
|  | Tianjin | / | / | / | / |
|  | Tibet | / | / | / | / |
|  | Xinjiang | / | / | / | / |
|  | Yunnan | / | / | 7.14 (8.89) | 7.14 (8.89) |
|  | Zhejiang | / | / | 9.31 (9.06) | **9.31 (9.06)** |
| Area | Urban | 3.27 (14.06) | 2.02 (6.80) | 1.16 (1.93) | **1.50 (4.03)** |
|  | Rural | 1.20 (2.14) | 0.74 (0.99) | 1.12 (3.14) | 0.99 (2.11) |
| Types of health facilities | Public | 1.24 (3.11) | 1.15 (4.26) | 1.45 (3.94) | 1.27 (4.04) |
|  | Private | 0.70 (0.90) | 2.53 (8.31) | 3.53 (7.38) | **2.24 (6.97)** |
| Levels of health facilities | Primary | 1.50 (4.17) | 1.02 (2.10) | 0.72 (2.54) | 1.12 (2.66) |
|  | Secondary | / | / | 1.00 (0.74) | 1.00 (0.74) |
|  | Tertiary | / | / | 1.00 (0.86) | 1.00 (0.86) |
|  | Tertiary & Secondary | / | 4.60 (1.66) | 3.17 (6.26) | **3.69 (5.17)** |
|  | Tertiary & Secondary& Primary | 0.82 (1.14) | 2.60 (9.89) | 2.44 (7.13) | 2.03 (7.63) |
| Characteristic (OB/LPG) | OB | 10.90 (17.30) | 14.23 (30.37) | 7.43 (10.00) | **10.29 (18.68)** |
|  | LPG | 0.80 (0.80) | 1.64 (4.57) | 1.04 (2.19) | 1.22 (3.03) |
| ATC | A | 1.60 (2.23) | 2.02 (7.50) | 1.52 (4.12) | 1.60 (4.71) |
|  | B | 2.01 (23.57) | 11.07 (54.32) | 7.34 (10.63) | **6.33 (17.15)** |
|  | C | 0.72 (7.46) | 4.46 (11.75) | 1.10 (4.13) | 2.49 (9.35) |
|  | D | 0.76 | 0.64 (0.02) | 1.67 | 0.64 (0.12) |
|  | G | / | 5.83 | 0.88 | 3.35 (4.95) |
|  | H | 0.48 (1.26) | 0.23 (0.43) | 0.81 (0.81) | 0.54 (1.18) |
|  | J | 1.14 (1.27) | 1.18 (3.05) | 1.43 (3.47) | 1.18 (2.66) |
|  | L | / | / | 1.00 (0.37) | 1.00 (0.37) |
|  | M | 1.82 (18.19) | 6.18 (19.20) | 3.26 (3.60) | 3.69 (17.84) |
|  | N | 0.49 (2.02) | 2.71 (10.69) | 1.85 (6.17) | 1.99 (6.57) |
|  | P | 7.92 (6.80) | 5.23 (4.67) | 4.47 (3.45) | 5.33 (5.93) |
|  | R | 0.83 (0.74) | 2.23 (14.21) | 4.05 (17.99) | 2.13 (10.62) |
|  | S | 0.16 | 0.14 | 0.24 | 0.16 (0.10) |
|  | V | / | / | 0.14 | 0.14 |
|  | Z | / | 4.47 (6.69) | 4.98 (9.29) | 4.53 (7.86) |

MPRs: median price ratios; IQR: interquartile range; OB: originator brand; LPG: lowest priced generic equivalent; ATC: anatomic therapeutic chemistry; A: alimentary tract and metabolism; B: blood and blood forming organs; C: cardiovascular system; D: dermatologicals; G: genito urinary system and sex hormones; H: systemic hormonal preparations, excl. sex hormones and insulins; J: antiinfectives for systemic use; L: antineoplastic and immunomodulating agents; M: musculoskeletal system; N: nervous system; P: antiparasitic products, insecticides and repellents; R: respiratory system; S: sensory organs; V: various; Z: unknow.

**Appendix 8. The median affordability of essential medicines in China [Median (IQR)]**

| Period | | Before 2009 | 2010-2014 | 2015-2019 | Overall |
| --- | --- | --- | --- | --- | --- |
| Overall | | 0.60 (2.20) | 0.80 (2.10) | 1.20 (3.13) | 0.88 (2.58) |
| Region | Eastern | 2.40 (5.30) | 0.70 (2.14) | 0.93 (2.56) | 0.82 (2.59) |
|  | Central | 0.40 (1.38) | 0.24 (0.76) | 1.58 (3.80) | 0.70 (2.49) |
|  | Western | 0.98 (1.39) | 1.30 (2.05) | 2.20 (3.66) | **1.40 (2.88)** |
|  | Northeastern | / | / | 0.17 (0.68) | 0.17 (0.68) |
| Province | Anhui | / | / | 0.37 (0.62) | 0.37 (0.62) |
|  | Beijing | / | 3.04 (3.24) | / | **3.04 (3.24)** |
|  | Chongqing | / | / | / | / |
|  | Fujian | / | / | / | / |
|  | Gansu | / | / | / | / |
|  | Guangdong | / | 0.38 (0.54) | / | 0.38 (0.54) |
|  | Guangxi | / | / | / | / |
|  | Guizhou | / | / | / | / |
|  | Hainan | / | / | / | / |
|  | Hebei | / | / | / | / |
|  | Henan | / | / | 0.72 (2.26) | 0.72 (2.26) |
|  | Heilongjiang | / | / | / | / |
|  | Hubei | 0.40 (1.30) | / | 2.09 (3.80) | 1.20 (3.00) |
|  | Hunan | / | / | / | / |
|  | Inner Mongolia | / | / | / | / |
|  | Jiangsu | / | 0.72 (2.95) | 1.88 (5.24) | 0.98 (3.22) |
|  | Jiangxi | / | / | / | / |
|  | Jilin | / | / | / | / |
|  | Liaoning | / | / | 0.17 (0.68) | 0.17 (0.68) |
|  | Ningxia | / | 0.61 (8.24) | / | 0.61 (8.24) |
|  | Qinghai | / | / | / | / |
|  | Shaanxi | / | 1.50 (3.10) | 2.20 (3.66) | 1.70 (3.40) |
|  | Shandong | 4.12 (7.38) | 0.32 (1.63) | / | 0.54 (4.77) |
|  | Shanghai | 1.90 (3.80) | 1.10 (1.73) | 0.94 (1.13) | 1.09 (1.77) |
|  | Shanxi | 0.26 (1.37) | 0.24 (0.76) | / | 0.24 (0.92) |
|  | Sichuan | 0.98 (1.39) | 0.70 (1.17) | / | 0.83 (1.17) |
|  | Tianjin | / | / | / | / |
|  | Tibet | / | / | / | / |
|  | Xinjiang | / | / | / | / |
|  | Yunnan | / | / | / | / |
|  | Zhejiang | / | 0.80 (1.70) | 0.80 (1.45) | 0.80 (1.50) |
| Area | Urban | 0.82 (3.13) | 0.80 (2.08) | 1.39 (3.19) | **0.95 (2.80)** |
|  | Rural | 0.70 (2.49) | 0.85 (2.88) | 0.82 (3.77) | 0.82 (3.10) |
| Types of health facilities | Public | 0.72 (2.49) | 0.70 (2.07) | 0.94 (3.25) | 0.80 (2.67) |
|  | Private | 0.50 (1.40) | 0.90 (2.00) | 1.80 (3.72) | **0.90 (2.30)** |
| Levels of health facilities | Primary | 0.61 (2.14) | 0.49 (1.43) | 1.06 (2.05) | 0.60 (1.82) |
|  | Secondary | / | / | 2.33 (2.14) | 2.33 (2.14) |
|  | Tertiary | / | 1.43 (2.99) | 2.00 (3.61) | 1.64 (3.10) |
|  | Tertiary & Secondary | / | 1.72 (57.07) | 4.26 (15.80) | **3.45 (20.39)** |
|  | Secondary & Primary | / | / | 0.72 (2.26) | 0.72 (2.26) |
|  | Tertiary & Secondary& Primary | 0.50 (2.20) | 0.87 (2.25) | 0.71 (2.69) | 0.74 (2.40) |
| Characteristic (OB/LPG) | OB | 3.45 (3.60) | 2.60 (7.25) | 3.24 (7.24) | **2.90 (6.68)** |
|  | LPG | 0.35 (0.65) | 0.40 (1.10) | 0.49 (1.55) | 0.40 (1.19) |
| ATC | A | 0.75 (1.70) | 0.60 (1.38) | 2.26 (3.60) | 1.40 (2.87) |
|  | B | 63.14 | 1.15 (2.78) | 3.78 (7.62) | 1.24 (3.54) |
|  | C | 0.21 (3.24) | 1.20 (3.30) | 0.73 (1.44) | 0.89 (2.39) |
|  | D | / | / | 0.17 (0.04) | 0.17 (0.04) |
|  | G | / | / | 0.17 | 0.17 |
|  | H | 0.44 | 0.03 (0.37) | 2.29 (4.80) | 0.41 (2.26) |
|  | J | 0.42 (1.30) | 0.41 (1.30) | 0.49 (1.18) | 0.47 (1.24) |
|  | L | / | 49.06 (154.17) | 3.36 (15.26) | **5.68 (56.47)** |
|  | M | 1.91 (4.58) | 1.40 (1.48) | 1.02 (2.27) | 1.31 (1.90) |
|  | N | 0.40 (3.07) | 0.40 (0.77) | 0.30 (2.49) | 0.39 (1.27) |
|  | P | 1.22 (0.69) | 0.64 (0.65) | 0.13 (0.21) | 0.74 (0.98) |
|  | R | 0.50 (1.20) | 0.61 (0.80) | 0.36 (2.78) | 0.60 (1.38) |
|  | V | / | / | 1.36 (1.27) | 1.36 (1.27) |
|  | Z | / | 1.44 (1.84) | 0.26 (0.64) | 0.97 (1.75) |

IQR: interquartile range; OB: originator brand; LPG: lowest priced generic equivalent; ATC: anatomic therapeutic chemistry; A: alimentary tract and metabolism; B: blood and blood forming organs; C: cardiovascular system; D: dermatologicals; G: genito urinary system and sex hormones; H: systemic hormonal preparations, excl. sex hormones and insulins; J: antiinfectives for systemic use; L: antineoplastic and immunomodulating agents; M: musculoskeletal system; N: nervous system; P: antiparasitic products, insecticides and repellents; R: respiratory system; V: various; Z: unknow.

**Appendix 9. PRISMA Checklist**

| **Section and Topic** | **Item #** | **Checklist item** | **Location where item is reported** |
| --- | --- | --- | --- |
| **TITLE** | | |  |
| Title | 1 | Identify the report as a systematic review. | P1 |
| **ABSTRACT** | | |  |
| Abstract | 2 | See the PRISMA 2020 for Abstracts checklist. | P1-2 |
| **INTRODUCTION** | | |  |
| Rationale | 3 | Describe the rationale for the review in the context of existing knowledge. | P2-P3 |
| Objectives | 4 | Provide an explicit statement of the objective(s) or question(s) the review addresses. | P3 |
| **METHODS** | | |  |
| Eligibility criteria | 5 | Specify the inclusion and exclusion criteria for the review and how studies were grouped for the syntheses. | P3 |
| Information sources | 6 | Specify all databases, registers, websites, organisations, reference lists and other sources searched or consulted to identify studies. Specify the date when each source was last searched or consulted. | P3 |
| Search strategy | 7 | Present the full search strategies for all databases, registers and websites, including any filters and limits used. | Appendix 1 |
| Selection process | 8 | Specify the methods used to decide whether a study met the inclusion criteria of the review, including how many reviewers screened each record and each report retrieved, whether they worked independently, and if applicable, details of automation tools used in the process. | P3 |
| Data collection process | 9 | Specify the methods used to collect data from reports, including how many reviewers collected data from each report, whether they worked independently, any processes for obtaining or confirming data from study investigators, and if applicable, details of automation tools used in the process. | P3-4 |
| Data items | 10a | List and define all outcomes for which data were sought. Specify whether all results that were compatible with each outcome domain in each study were sought (e.g. for all measures, time points, analyses), and if not, the methods used to decide which results to collect. | P3-4 |
|  | 10b | List and define all other variables for which data were sought (e.g. participant and intervention characteristics, funding sources). Describe any assumptions made about any missing or unclear information. | P3-4, Appendix 2 |
| Study risk of bias assessment | 11 | Specify the methods used to assess risk of bias in the included studies, including details of the tool(s) used, how many reviewers assessed each study and whether they worked independently, and if applicable, details of automation tools used in the process. | P4 |
| Effect measures | 12 | Specify for each outcome the effect measure(s) (e.g. risk ratio, mean difference) used in the synthesis or presentation of results. | P4 |
| Synthesis methods | 13a | Describe the processes used to decide which studies were eligible for each synthesis (e.g. tabulating the study intervention characteristics and comparing against the planned groups for each synthesis (item #5)). | P4 |
|  | 13b | Describe any methods required to prepare the data for presentation or synthesis, such as handling of missing summary statistics, or data conversions. | NA |
|  | 13c | Describe any methods used to tabulate or visually display results of individual studies and syntheses. | NA |
|  | 13d | Describe any methods used to synthesize results and provide a rationale for the choice(s). If meta-analysis was performed, describe the model(s), method(s) to identify the presence and extent of statistical heterogeneity, and software package(s) used. | P4 |
|  | 13e | Describe any methods used to explore possible causes of heterogeneity among study results (e.g. subgroup analysis, meta-regression). | P4 |
|  | 13f | Describe any sensitivity analyses conducted to assess robustness of the synthesized results. | NA |
| Reporting bias assessment | 14 | Describe any methods used to assess risk of bias due to missing results in a synthesis (arising from reporting biases). | NA |
| Certainty assessment | 15 | Describe any methods used to assess certainty (or confidence) in the body of evidence for an outcome. | NA |
| **RESULTS** | | |  |
| Study selection | 16a | Describe the results of the search and selection process, from the number of records identified in the search to the number of studies included in the review, ideally using a flow diagram. | P4 |
|  | 16b | Cite studies that might appear to meet the inclusion criteria, but which were excluded, and explain why they were excluded. | Appendix 3 |
| Study characteristics | 17 | Cite each included study and present its characteristics. | P4-5, Appendix 4 |
| Risk of bias in studies | 18 | Present assessments of risk of bias for each included study. | P4-5, Appendix 6 |
| Results of individual studies | 19 | For all outcomes, present, for each study: (a) summary statistics for each group (where appropriate) and (b) an effect estimate and its precision (e.g. confidence/credible interval), ideally using structured tables or plots. | NA |
| Results of syntheses | 20a | For each synthesis, briefly summarise the characteristics and risk of bias among contributing studies. | P5 |
|  | 20b | Present results of all statistical syntheses conducted. If meta-analysis was done, present for each the summary estimate and its precision (e.g. confidence/credible interval) and measures of statistical heterogeneity. If comparing groups, describe the direction of the effect. | P5, Appendix 7,8 |
|  | 20c | Present results of all investigations of possible causes of heterogeneity among study results. | NA |
|  | 20d | Present results of all sensitivity analyses conducted to assess the robustness of the synthesized results. | NA |
| Reporting biases | 21 | Present assessments of risk of bias due to missing results (arising from reporting biases) for each synthesis assessed. | NA |
| Certainty of evidence | 22 | Present assessments of certainty (or confidence) in the body of evidence for each outcome assessed. | NA |
| **DISCUSSION** | | |  |
| Discussion | 23a | Provide a general interpretation of the results in the context of other evidence. | P5-P8 |
|  | 23b | Discuss any limitations of the evidence included in the review. | P7 |
|  | 23c | Discuss any limitations of the review processes used. | P7 |
|  | 23d | Discuss implications of the results for practice, policy, and future research. | P7-8 |
| **OTHER INFORMATION** | | |  |
| Registration and protocol | 24a | Provide registration information for the review, including register name and registration number, or state that the review was not registered. | P3 |
|  | 24b | Indicate where the review protocol can be accessed, or state that a protocol was not prepared. | P3 |
|  | 24c | Describe and explain any amendments to information provided at registration or in the protocol. | NA |
| Support | 25 | Describe sources of financial or non-financial support for the review, and the role of the funders or sponsors in the review. | P8 |
| Competing interests | 26 | Declare any competing interests of review authors. | P8 |
| Availability of data, code and other materials | 27 | Report which of the following are publicly available and where they can be found: template data collection forms; data extracted from included studies; data used for all analyses; analytic code; any other materials used in the review. | P8 |

**Appendix 10. Reference list for included studies**

^1-65^

1. Wang L, Dai L, Liu H, Dai H, Li X, Ge W. Availability, affordability and price components of insulin products in different-level hospital pharmacies: Evidence from two cross-sectional surveys in Nanjing, China. *PLoS One* 2021; 16: e0255742.
2. Li Z, Feng Q, Kabba JA, et al. Prices, availability and affordability of insulin products: a cross-sectional survey in Shaanxi Province, western China. *Tropical medicine & international health : TM & IH* 2019; 24: 43-52.
3. Wang H, Sun Q, Vitry A, Nguyen TA. Availability, Price, and Affordability of Selected Essential Medicines for Chronic Diseases in 11 Countries of the Asia Pacific Region: A Secondary Analysis. *Asia-Pacific journal of public health* 2017; 29: 268-77.
4. Jiang M, Zhou Z, Wu L, et al. Medicine prices, availability, and affordability in the Shaanxi Province in China: implications for the future. *International journal of clinical pharmacy* 2015; 37: 12-7.
5. Wang X, Fang Y, Yang S, et al. Access to paediatric essential medicines: a survey of prices, availability, affordability and price components in Shaanxi Province, China. *PLoS One* 2014; 9: e90365.
6. Yang H, Dib HH, Zhu MM, Qi G, Zhang XP. Prices, availability and affordability of essential medicines in rural areas of Hubei Province, China. *Health Policy And Planning* 2010; 25: 219-29.
7. Xu R, Li S, Lv X, Xie X. Prices, availability, and affordability of national essential medicines in public primary hospitals: A cross-sectional survey in poverty-stricken rural areas in China. *The International journal of health planning and management* 2020; 35: 545-57.
8. Yang C, Hu S, Zhu Y, Zhu W, Li Z, Fang Y. Evaluating access to oral anti-diabetic medicines: A cross-sectional survey of prices, availability and affordability in Shaanxi Province, Western China. *PLoS One* 2019; 14: e0223769.
9. Guan X, Hu H, Man C, Shi L. A survey of availability, price and affordability of essential medicines from 2011 to 2016 in Chinese secondary and tertiary hospitals. *International journal for equity in health* 2018; 17: 158.
10. Yang C, Hu S, Ye D, Jiang M, Babar ZU, Fang Y. Evaluating Price and Availability of Essential Medicines in China: A Mixed Cross-Sectional and Longitudinal Study. *Frontiers in pharmacology* 2020; 11: 602421.
11. Chen C, Feng Z, Ding Y, et al. What Factors Hindered the Access to Essential Anticancer Medicine in Public Hospitals for the Local Population in Hubei Province, China. *Frontiers in pharmacology* 2021; 12: 734637.
12. Dong Z, Tao Q, Yan B, Sun G. Availability, prices and affordability of essential medicines in Zhejiang Province, China. *PLoS One* 2020; 15: e0241761.
13. Gong S, Cai H, Ding Y, et al. The availability, price and affordability of antidiabetic drugs in Hubei province, China. *Health policy and planning* 2018; 33: 937-47.
14. Zhu Y, Wang Y, Sun X, Li X. Availability, Price and Affordability of Anticancer Medicines: Evidence from Two Cross-Sectional Surveys in the Jiangsu Province, China. *International journal of environmental research and public health* 2019; 16.
15. Song Y, Bian Y, Zhen T. Making medicines more accessible in China: An empirical study investigating the early progress of essential medicine system. *PLoS ONE* 2018; 13: e0201582.
16. Liu C, Zhang X, Liu C, Ewen M, Zhang Z, Liu G. Insulin prices, availability and affordability: a cross-sectional survey of pharmacies in Hubei Province, China. *BMC health services research* 2017; 17: 597.
17. Xi X, Li W, Li J, et al. A survey of the availability, prices and affordability of essential medicines in Jiangsu Province, China. *BMC health services research* 2015; 15: 345.
18. Zhu Y, Xu X, Fang W, Wang Y, Dai H, Li X. Availability, cost and affordability of selected antibiotics and antiviral medicines against infectious diseases from 2013 to 2019 in Nanjing, China. *Tropical medicine & international health : TM & IH* 2021; 26: 518-29.
19. Sun X, Wei J, Yao Y, et al. Availability, prices and affordability of essential medicines for children: a cross-sectional survey in Jiangsu Province, China. *BMJ Open* 2018; 8: e023646.
20. Wu G, Gong S, Cai H, Ding Y. The availability, price and affordability of essential antibacterials in Hubei province, China. *BMC health services research* 2018; 18: 1013.
21. Ming Y. Study on The Price Level And Availability of Essential Medicines in Jilin Province [Chinese]: Jilin University; 2016.
22. Chen C, Lu Y, Ai D, Wu T. Analysis of the Accessibility of Common Essential Medicine for 6 Kinds of Chronic Disease in Primary Health Care Institutions in Hubei Province from 2015 to 2017 [Chinese]. *China Pharmacy* 2019; 30: 5-10.
23. Chen L, Zhang M, Xu X, Guo L. A comparison analysis on drug price and sales volume before and after implementation of national essential medicines system in Fujian Province [Chinese]. *Chinese Journal of Health Policy* 2011; 4: 7-12.
24. Dai T, Chen Y, Bai B. Effect on implementing essential medicine centralized bidding and purchasing policy [Chinese]. *Chinese Journal of Health Policy* 2013; 6: 19-25.
25. Dai Y, Li Z, Xu H, et al. A multicenter survey of the accessibility of essential medicines for children in China [Chinese]. *Chin J Pediatr* 2020; 58: 301-7.
26. Dou Z, Wang Q. Analysis and Investigation on Application of Oral Antihypertensive Drugs in 5 County Public Medical Institutions of Luohe in 2015 [Chinese]. *Evaluation and Analysis of Drug-Use in Hospitals of China* 2017; 17: 1399-401.
27. Guan X, Li H, Liu Y, Shi L. Empirical Study on Essential Medicine Price in China [Chinese]. *China Pharmacy* 2013; 24: 2224-8.
28. Guan X, Xin X, Liu Y, Shi L. Empirical Study on Affordability of Essential Medicine in China [Chinese]. *China Pharmacy* 2013; 24: 2220-4.
29. Jiang M, Wang L, Wang W, et al. Comparative Study on the Price and Availability of Essential Drugs in Public Hospital and Retail Pharmacy of Shaanxi Province [Chinese]. *China Pharmacy* 2013; 24: 308-13.
30. Jiang M, Zhou Z, Fang Y, et al. Comparative Study on the Availability of Drugs in Public Hospitals and Retail Pharmacies in Shaanxi Province [Chinese]. *Chinese Journal of Health Policy* 2013; 6: 37-42.
31. Jiang X, Wang Y, Jia S, Gao Y, Sun L. Evaluation of Essential Medicines Accessibility in Liaoning Province [Chinese]. *Chinese Pharmaceutical Journal* 2019; 54: 501-5.
32. Jiang M, Lou H, Sun Q, Wang H. Price and the accessibility of essential medicines for the elderly with chronic diseases in the Asia Pacific Region [Chinese]. *Journal of Shandong University(Health Sciences)* 2017; 55: 80-4.
33. Li F. Availability of Essential Medicines Investigation of Community Health Services in Guangzhou [Chinese]. *Chinese Health Economics* 2011; 30: 52-4.
34. Li L, Sun L. Analysis of the Application of National Essential Medicines in Our Hospital during 2010-2011 [Chinese]. *China Pharmacy* 2013; 24: 3050-3.
35. Li P. Study on Availability of Enssential Medicines among Counties and Towns in Hubei Province [Chinese]: Huazhong University of Science and Technology; 2009.
36. Zhang Y, Li X. Investigation and Analysis of Availability and Affordability of Essential Medicine in Nanjing Based on WHO/HAI Standard Survey Method [Chinese]. *China Pharmacy* 2015; 26: 4188-92.
37. Zhang Y, Chang Y. Empirical study on the affordability of essential medicines in some provinces and cities in western China [Chinese]. *Guide of China Medicine* 2013; 11: 676-7.
38. Zhang X, Shao Q. Analysis of the Utilization of Essential Medicines in Outpatient Department of a Women and Children’s Hospital during 2012-2013 [Chinese]. *China Pharmacy* 2015; 26: 2498-500.
39. Li W, Li Q, Shao Y, et al. Changes of Drug Economic Burden of Patients in Shanghai after Implementation of National Essential Drug System [Chinese]. *China Pharmacy* 2012; 23: 4135-7.
40. Li X, Zhang Y, Wu S, Liang H. An evidence-base study on the affordablity of essential drugs in Essential Medicine System [Chinese]. *Chinese Rural Health Service Administration* 2013; 33: 10-3.
41. Lin T, Hu M, Wu J. National essential medicine system in primary medical institutions in rural Sichuan [Chinese]. *Chinese Journal of Health Policy* 2013; 6: 48-53.
42. Ma R. Research on Enhancing the Welfare Effect of National Essential Drug System Based on HRQOL [Chinese]: Nanjing University Of Chinese Medicine; 2017.
43. Shang J, Guo Z, Lin Q, et al. Analysis of accessibility of essential medicine in Beijing [Chinese]. *Chinese Journal of Health Policy* 2016; 9: 52-8.
44. Shen Y, Wan Q, Xie N, Xu Q. Empirical research on the affordability of the antihypertensive drugs in the basic drug list from Qingpu District [Chinese]. *Journal of Pharmaceutical Practice* 2018; 36: 536-40.
45. Shen Y, Wang Y, Xie N, Wan Q, Li H, Tang K. Empirical research on the price level of drugs for non-communicable diseases in basic drug list of Shanghai Qingpu District [Chinese]. *Pharmaceutical Care and Research* 2017; 17: 298-301.
46. Song Y, Bian Y. Empirical study of China’s essential medicine system on improving access to medicines [Chinese]. *Chinese Journal of Health Policy* 2012; 5: 16-20.
47. Tang S, Hou T, Zhao Q, Du J, Yin S, Bian Y. Price and Affordability of Essential Medicine in a County Hospital from Shanxi Province [Chinese]. *China Pharmacy* 2014; 25: 2223-6.
48. Wang So, Liu Y, Guan X. Empirical study on the essential drugs′ price in parts of China [Chinese]. *Chinese Health Service Management* 2011; 28: 266-7+309.
49. Wang X, Yang S, Fang Y, Jiang M, Wu L. Study on the Availability and Price Pediatric Essential Medicines in Retail Pharmacies in Shaanxi Province Using WHO/HAI Methodology [Chinese]. *China Pharmacy* 2014; 25: 678-81.
50. Wang X, Yang S, Fang Y, Jiang M, Wu L. Study on the Availability and Price of Pediatric Essential Medicines in Public Hospitals in Shaanxi Province Using WHO/HAI Methodology [Chinese]. *China Pharmacy* 2014; 25: 681-4.
51. Wang X, Zhang A, Wang H, Xu D. Evaluation Study on the Availability and Prices of Paediatric Essential Drugs in Weifang [Chinese]. *Chinese Pharmaceutical Affairs* 2020; 34: 1085-92.
52. Wei G, Wang X, Li X, Li L, Chen J, Shi L. A Surveyon the availability, price and affordability of essential medicine for children in Eastern China [Chinese]. *Chinese Journal of Health Policy* 2019; 12: 72-8.
53. Wei Y, Yin W, Ma X, et al. Analyzing the Impact of Essential Medicine System on the Affordability of the Medicines in Township Hospitals in Shandong [Chinese]. *Chinese Health Economics* 2013; 32: 18-20.
54. Xu W, Yin D. Empirical Study on the Affordability of Essential Drugs in China [Chinese]. *China Pharmacy* 2012; 23: 3745-7.
55. Xu W, Yin D. Evidence-base study on the affordability of essential medicines in Jiangsu Province [Chinese]. *Shanghai Medical ＆ Pharmaceutical Journal* 2012; 33: 43-7.
56. Yang H. Study on Availability of Enssential Medicines among Counties and Towns in Hubei Provinc [Chinese]: Huazhong University of Science and Technology; 2009.
57. Yang H. The Study on the Accessibility of Essential Drugs in the Rural Areas of Shandong Province [Chinese]: Shandong University; 2012.
58. Li F. Availability of Essential Medicines Research of Community Health Services in Guangzhou [Chinese]: Guangzhou University of Chinese Medicine; 2011.
59. Wang So, Liu Y, Guan X, Shi L. Empirical Study of the Affordability of Essential Medicines in the Regional Provinces of China [Chinese]. *Drug Evaluation* 2011; 8: 33-7.
60. Yang H, Li P, Zhang X. Procurability to Essential Medicines in Rural Area of Hubei Xiaogan Rural Area [Chinese]. *China Pharmacy* 2008: 2244-7.
61. Ye L. Study on National Essential Medicine Policy in China [Chinese]: Fudan University; 2008.
62. Yin S, Bian Y. Analysis of Drug Use,Price Levels and Affordability of a County-level Public Hospital in Northwestern Area [Chinese]. *China Pharmacy* 2013; 24: 1465-7.
63. Zhang L, Zhou Q, Zhang X, Yu Y, Wang S, Shao R. Investigation and Study on the Accessibility of Antibiotics in Essential Medicine List in Medical Institutions of Nanjing Area [Chinese]. *China Pharmacy* 2020; 31: 1281-7.
64. Zhang M. Managing and Analyzing the Implementation of Essential Medicine System in Fujian Province by Using Project Software and Media Price Ratio [Chinese]: Fujian Medical University; 2012.
65. Zhang P, Chen Y. Comparative study on the price of antimicrobial drugs in primary medical institutions in China [Chinese]. *Modern Business Trade Industry* 2013; 25: 45-6.
